# Diversifying Selection on Flavanone 3-Hydroxylase and Isoflavone Synthase Genes in Cultivated Soybean and Its Wild Progenitors

**DOI:** 10.1371/journal.pone.0054154

**Published:** 2013-01-16

**Authors:** Hao Cheng, Jiao Wang, Shanshan Chu, Hong-Lang Yan, Deyue Yu

**Affiliations:** National Center for Soybean Improvement, National Key Laboratory of Crop Genetics and Germplasm Enhancement, Nanjing Agricultural University, Nanjing, China; University College London, United Kingdom

## Abstract

Soybean isoflavone synthase (IFS) and flavanone 3-hydroxylase (F3H) are two key enzymes catalyzing the biosynthesis of isoflavonoids and flavonoids, both of which play diverse roles in stress responses. However, little is known about the evolutionary pattern of these genes in cultivated soybean and its wild progenitors. Herein, we investigated the nucleotide polymorphisms in Isoflavone synthase (*IFS1*, *IFS2*) and Flavanone 3-hydroxylase (*F3H2*) genes from 33 soybean accessions, including 17 cultivars (*Glycine max*) and 16 their wild progenitors (*Glycine soja*). Our data showed that the target genes shared the levels of nucleotide polymorphism with three reference genes involved in plant-microbe interactions, but possessed a much higher nucleotide polymorphism than other reference genes. Moreover, no significant genetic differentiation was found between cultivated soybean and its wild relatives in three target genes, despite of considering bottleneck and founder effect during domestication. These results indicate that *IFS* and *F3H* genes could have experienced gene introgressions or diversifying selection events during domestication process. Especially, *F3H2* gene appears to evolve under positive selection and enjoy a faster evolutionary rate than *IFS1* and *IFS2* genes.

## Introduction

Cultivated soybean [*Glycine max* (L.) Merr.] was domesticated from its annual wild relative [*Glycine soja* Sieb. and Zucc.] in East Asia more than 3,000 years ago [1], [2]. Recent research showed that cultivated soybean has lost many rare sequence variants in wild soybean and has undergone numerous allele frequency changes throughout its cultivated history, indicating a severe genetic bottleneck during domestication [3]. Therefore, wild soybean could serve as a vast genetic reservoir and invaluable germplasm resource for both broadening genetic diversity and improving important agronomic traits in soybean breeding.

Soybean isoflavone synthase (IFS) and flavanone 3-hydroxylase (F3H) genes encode two key enzymes involved in the phenylpropanoid pathway (see Figure S1). Phenylpropanoid products, isoflavonoids and flavonoids, play diverse roles in the responses of plants to different biotic and abiotic stresses, particularly in plant-environment interactions [4]. In all cases the unique aryl migration reaction to create isoflavones is mediated by IFS, a legume specific enzyme. IFS enzyme belongs to the CYP93C subfamily of cytochrome P450 monooxygenases and its encoding gene has been identified [5]–[7]. There were two copies of IFS genes in soybean genome, *IFS1* and *IFS2*, both of which function in isoflavones synthesis. The flavanones in this reaction, including naringenin and liquiritigenin, are also substrates for various other flavonoid biosynthesis processes. The most common enzyme competing with IFS for these common substrates is flavanone 3-hydroxylase (F3H), a 2-oxoglutartate-dependent dioxygenase that initiates syntheses of the majority of flavonoid compounds, including flavonols, anthocyanins, and proanthocyanidins [8]. There were also two tandem duplicates *F3H1* and *F3H2* in soybean, all of which might contribute to isoflavone accumulation in soybean seeds [9], [10]. In addition, soybean isoflavones are associated with many health benefits of soy consumption. Because of their biological importance, the genes of isoflavone synthase i.e., *IFS1*, *IFS2* and flavanone 3-hydroxylase *F3H2* gene, were chosen as target genes in this study.

The related genes of IFS and F3H enzyme have been decoded and their DNA sequences have been investigated. As for IFS genes, *IFS1* and *IFS2* genes from 18 Korean soybeans were isolated and sequenced [11]. Variations at the amino acid level among the isoflavone synthases (IFS1, IFS2) were uncovered, yet the detailed nucleotide diversity and selection information were not presented. Turning to *F3H* gene, Aguadé et al (2001) surveyed the pattern of nucleotide variation at *F3H* gene by sequencing a sample of 20 worldwide *Arabidopsis thaliana* ecotypes with one *Arabidopsis lyrata* spp. *petraea* stock as outgroup. They found that *F3H* gene in *Arabidopsis* was compatible with a neutral model with no recombination [12]. However, so far there is no parallel study of nucleotide polymorphisms in soybean *F3H* gene as to the knowledge of the authors.

As functionally important and environment response related genes, *IFS1*, *IFS2* and *F3H2* could show the same or a different evolutionary pattern from other functional genes. Analyzing patterns of DNA polymorphisms and interspecific divergence of a given gene can offer important messages for elucidating the selective forces acting on this gene due to its functional requirement. Comparative investigation of polymorphism between wild and cultivate soybean could provide the evidence for the genetic variation origin and maintenance. Therefore, in our study, we investigated the level and pattern of diversity along the sequences of *IFS* and *F3H* genes in both cultivated and wild soybean accessions. Our results revealed that the three target genes expressed consistent evolutionary pattern with environment response related genes, with much higher level of nucleotide polymorphism than the other reference genes. Particularly, *F3H2* gene was under positive selection and evolved at a faster rate than *IFS* genes. In summary, *F3H* gene and *IFS* genes respond to diverse environments and could be applied for the association analyses of SNPs corresponding to various stress.

## Results

### Patterns of Polymorphism in Target and Reference Genes

Several genetic estimators, including π, *H_d_*_,_ θ, *θ_sil_* and *K_sil_* etc, were employed to investigated the patterns of polymorphism in both target and reference genes. Overall, our results consistently suggest a distinctive evolution pattern in *IFS1*, *IFS2* and *F3H2* genes during soybean domestication compared with the reference genes (Table 1).

**Table 1:** Nucleotide variation at *IFS1*, *IFS2*, *F3H* genes and reference genes, only CDS were considered.

Gene	π (%)		Hd		θ (%)		θ_sil_ (%)		K_sil_(%)	Dxy(%)
	C	W	C	W	C	W	C	W		
*IFS1*	0.21	0.23	0.97	0.97	0.32	0.5	0.81	1.07	0.59	0.23
*IFS2*	0.16	0.17	0.79	0.97	0.4	0.39	0.9	0.49	0.32	0.17
*F3H2*	0.95	0.91	0.84	0.92	1	0.77	0.67	0.9	0.92	1
Average	0.44	0.44	0.87	0.95	0.57	0.55	0.79	0.82	0.61	0.47
R1										
EU450800	0.27	0.41	1.00	0.99	0.40	0.66	0.55	1.07	0.48	0.35
L20310	0.43	0.51	0.87	0.92	0.41	0.50	0.22	0.43	0.20	0.46
D13505	0.24	0.18	0.98	0.93	0.35	0.31	1.13	0.73	0.64	0.22
Average	0.32	0.37	0.95	0.95	0.39	0.49	0.64	0.74	0.44	0.34
R2										
K00821	0	0.05	0	0.33	0	0.10	0	0.14	0.06	*0.03*
AF083880	0	0.01	0	0.12	0	0.03	0	0	0	0.01
AF124148	0.16	0.10	0.91	0.66	0.12	0.12	0.23	0.22	0.26	0.14
U13987	0	0.08	0	0.52	0	0.11	0	0.23	0.08	0.04
E00532	0.05	0.05	0.25	0.22	0.07	0.06	0	0	0	0.17
L10292	0.05	0.07	0.34	0.42	0.04	0.08	0	0.16	0.07	0.06
AB030491	0	0.03	0	0.32	0	0.06	0	0	0	0.02
AF079058	0.04	0	0.42	0	0.03	0	0.11	0	0.11	0.02
D31700	0.04	0.03	0.13	0.23	0.08	0.08	0.37	0.35	0.14	0.03
J01298	0.06	0.14	0.59	0.82	0.11	0.13	0.12	0.44	0.27	0.11
AF089850	0.12	0.07	0.39	0.17	0.08	0.15	0.29	0.29	0.35	0.10
M94012	0.11	0.12	0.52	0.41	0.05	0.15	0	0	0	0.15
AB004062	0.04	0.21	0.18	0.66	0.06	0.12	0	0	0	0.18
M11317	0.28	0.21	0.65	0.56	0.19	0.38	0.32	0.64	0.36	0.25
Average	0.07	0.08	0.31	0.39	0.06	0.11	0.10	0.18	0.12	0.09

R1 represents plant-environment interaction related reference genes, which include EU450800, the disease resistance gene *Rps1-k-1*; L20310, nodulin (nod-20) gene and D13505, early nodulin related gene; R2 signifies other fourteen reference genes.

C, Cultivated soybean; W, Wild soybean.

π, Nucleotide diversity with Jukes and Cantor correction.

Hd, Haplotype diversity.

θ, Watterson’s estimator of θ per basepair calculated on the total number of polymorphic sites.

θ_sil_, Watterson’s estimator of θ per basepair calculated on the silent sites.

K_sil_, Average divergence per silent site (with the Jukes and Cantor correction) between cultivars and wild soybean.

*Dxy*, Nucleotide divergence with Jukes and Cantor correction between cultivars and wild soybean.

Data in table 1 showed that the average nucleotide diversities (π) of the target genes (0.44% in cultivated soybean, 0.44% in wild soybean) were similar to those (0.32% in cultivated soybean, 0.37% in wild soybean) of R1 genes in both cultivated and wild soybeans, while more than 5 fold higher than those in R2 genes (0.07% in wild soybean, 0.08% in wild soybean). The target genes were known to be involved in response to various environmental stresses [4]. And R1 genes play roles in plant-microbe interaction. Therefore, we analyzed these genes together as environmental response genes. Obviously, environmental response genes have higher nucleotide diversities than those in R2 genes in both cultivated and wild soybeans (*t*-test, *P*<0.05). Similarly, this result was also observed when using haplotype diversity (*H_d_*) as a estimator, regardless of using the cultivated or wild samples (Table 1). These results indicate that more abundant polymorphisms were maintained in the environmental response genes than other genes, which might be due to diversify selection acting on these genes.

In addition, we compared the values of *θ* (nucleotide diversity per sequence calculated based on the number of segregating sites) between cultivar and wild soybeans. The values of *θ* in cultivated soybeans were significantly lower than those of wild soybeans for R2 genes, which were only 54.5% of the levels in their wild relatives (t-test, *P*<0.01). It indicates the existence of bottleneck and founder effect in soybean genomes during the domestication process. In contrast, the levels of polymorphism in cultivars were 92.1% (*θ*) of those found in wild soybean for the target and R1 genes, reflecting the introgression or diversifying selection effect on these environmental response genes to respond to changes of the environment. All these results also indicate that *IFS* and *F3H* genes have a distinctive evolutionary pattern during soybean domestication.

To further investigate this interesting contrast, intra-specific silent diversity (*θ_sil_*) was employed in our analysis. Under neutral evolution model, it is expected that intra-specific silent diversity (*θ_sil_*) and inter-specific silent divergence (*K_sil_*) are correlated with each other. In both cultivated and wild soybeans, we found significant positive correlations between *θ_sil_* and *K_sil_* in R2 genes (*P*<0.01). However, the correlation was undetectable in environmental response genes, also suggesting different evolutionary pattern involved in these environmental response genes.

### High Nucleotide Polymorphim in F3H2 Gene

**Figure 1:**
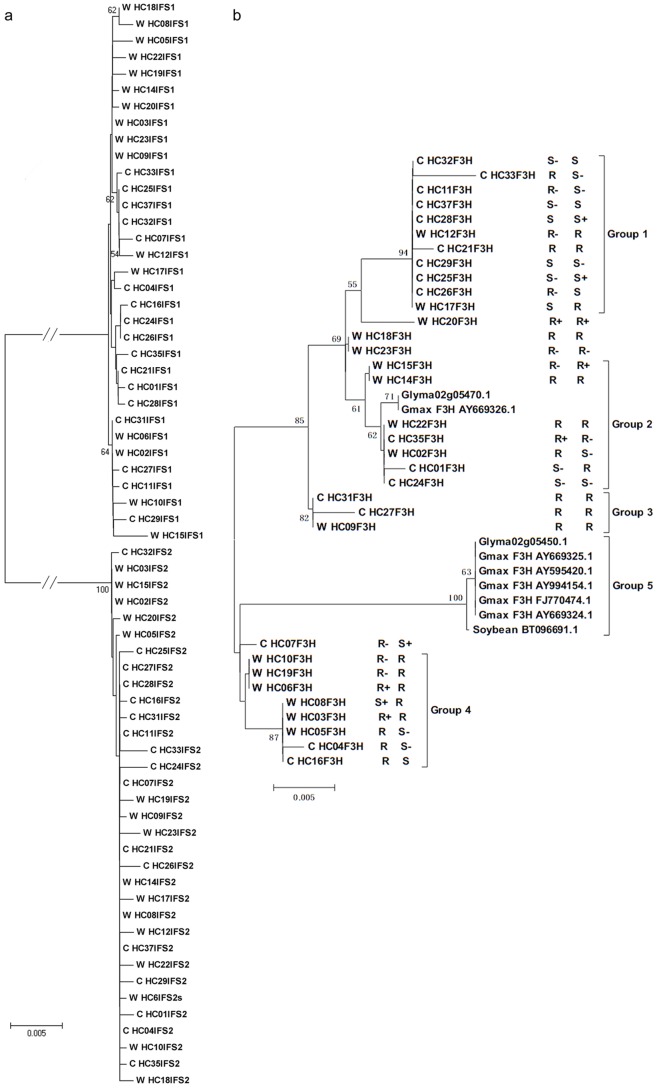
Phylogenetic trees of *IFS1* and *IFS2* (a), *F3H2* (b). The trees were estimated by neighbor-joining (NJ) method based on multiple CDS sequence alignments. CDS of *F3H1* (Glyma02g05450.1) alleles were used as outgroup for soybean *F3H2* in this study. The synthetic disease index (SDI) [32] was used in evaluation of soybean resistance to soybean mosaic virus. If the SDI was under 0, 20 and 35, the accession was classified as R+, R and R-. Meanwhile, we defined the accession as S+, S and S- respectively, if the SDI was above 70, 51 and 36.The resistance responses of 33 soybean accessions to SMV strains SC-3 and SC-7 were listed on the former and latter columns respectively. Bootstrap values >50% are indicated on the branches.

Among the three target genes, *F3H2* has the highest nucleotide diversity (0.91–0.95%), which is four to five fold higher than that in *IFS1* and *IFS2* genes (0.16–0.23%). Meanwhile, the value of nucleotide divergence (*Dxy*) between wild and cultivated soybean was also much higher in *F3H2* (1.00%) than that of *IFS* genes (0.17–0.23%). Given the same divergence time between cultivated and wild soybeans, *F3H2* genes must enjoy a much higher evolutionary rate. The phylogenetic tree of *F3H2* alleles was constructed using *F3H1* as an outgroup (Figure 1b). Using a cutoff of nucleotide-divergence value 0.008 and significant genetic differentiation between each group (Table S5), we revealed five major groups of *F3H* alleles (Figure 1b). According to the results, the nucleotide diversity within each group ranged from 0.048% to 0.264%, much lower than nucleotide divergence between each two groups (0.813% to 3.502%). Spontaneously, it is reasonable to assume that these grouping might be the result of demographic factors. However, the facts that the alleles from same population scattered in different clades of the phylogenetic tree and the alleles in the same clade contain individuals from different populations reject the hypothesis. On the other hand, the phylogenetic tree showed that group 4 was apparently an intermediate group locating between *F3H1* and *F3H2* clades.

**Figure 2:**
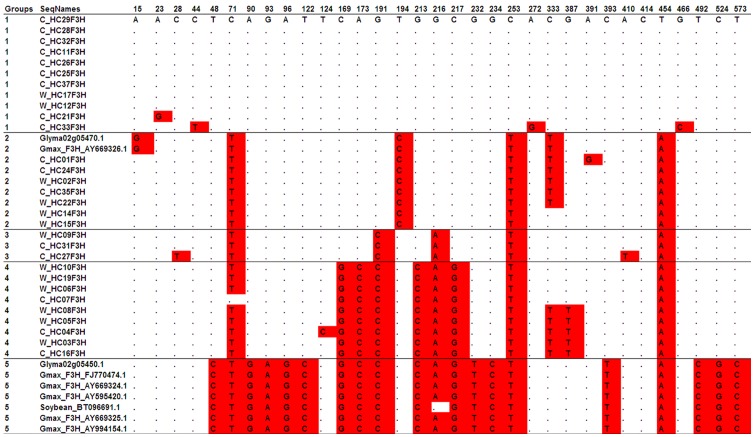
Nucleotide polymorphism sites of *F3H*2 genes. The locations of the polymorphism sites were shown in the above line. The polymorphic sites were highlighted by red color.

Subsequently, we examine the distribution pattern of polymorphism sites along *IFS1* (Figure S2a), *IFS2* (Figure S2b) and *F3H2* (Figure 2). According to our results, distinct polymorphism distribution patterns displayed among these three genes. In *IFS1* and *IFS2* genes, most of the polymorphism sites were singletons scattered across the coding sequences of genes, although four non-linked parsimony informative sites detected in *IFS1* gene. In contrast, *F3H2* gene (Figure 2) presents a different polymorphism pattern from two *IFS* genes. We found several apparent groups of linked polymorphism sites across the whole gene region. Most of these groups cover majorly cultivated or wild soybean varieties. Especially, an intermediate group between *F3H1* and *F3H2* was detected, sharing nucleotide sequences with both copies. These phenomena provided further evidence that genetic introgression or sequence exchange exist not only between wild and cultivated soybean but also among paralogs. In addition, there was no clear relationship between patterns of polymorphism sites and areas where those soybean samples come from, suggesting that gene introgression or recombination frequently happened at this locus.

Furthermore, our previous study has shown that single nucleotide polymorphisms were associated with SMV strain resistance for *F3H2* locus [13]. Therefore, we combined the phenotypes for each soybean accessions to SMV strains SC-3 and SC-7 with the phylogenetic tree of *F3H2*. Both resistance and susceptible phenotypes were discovered in each group, suggesting diverse resistance responses were preserved at this locus. Moreover, the resistance rates for each group were defined as the number of resistance accessions to the total number of accessions. The resistance rate was lowest in group 1 (36.4%) comparing to those values in group 2, 3 and 4, ranging from 71.4–100%. These suggest accessions in group 1 were more susceptible to SMVs.

### Genetic Structure and Differentiation between Cultivated and Wild Soybean

To clarify the phylogenetic relationship of the three genes between cultivated and wild soybean, NJ trees were constructed based on nucleotide variations (Figure 1a, 1b). In the phylogenetic trees of *IFS1* and *IFS2* (Figure 1a), and *F3H2* (Figure 1b) genes, cultivated and wild soybean were not clearly separated to form different clades. Instead, there were always some mixed clades including homolog genes from both cultivars and their wild relatives (Figure 1a, 1b), suggesting that there is no apparent differentiation in these three genes between cultivated and wild soybeans. For the 17 reference genes, the mixtures between the cultivated and wild soybeans can also be detected from their phylogenetic trees (see Figure S3). Meanwhile, the nucleotide divergence (*Dxy*) of three target genes and the reference genes between cultivated and wild soybean are similar to the intra-specific nucleotide diversity (π) in wild soybean (Table 1). Besides, there were no fixed polymorphisms but a much larger number of shared polymorphisms detected between cultivated and wild soybeans (Table 2).

**Table 2:** Genetic differentiation between cultivated and wild soybean at *IFS1*, *IFS2*, *F3H* genes and reference genes, only CDS were considered.

Gene	Polymorphisms between C and W				R_M_			Genetic differentiation between W and C				
	Fixed	In C	In W	Shared	C	W	All	S_nn_	F_st_	Chi-square statistic		
										χ ^2^	df	P-value
*IFS1*	0	14	23	3	1	3	3	0.78**	0.05*	30.33	25	0.21
*IFS2*	0	22	20	0	0	2	2	0.57**	0.01	26.59	21	0.18
*F3H2*	0	8	3	12	2	5	5	0.60	0.07	17.76	13	0.17
Average	0	14.33	15.33	5.00	1.00	3.33	3.33					
R1												
EU450800	0	17	52	31	8	13	20	0.45	0.01	29.32	29	0.45
L20310	0	0	2	8	2	4	4	0.40	−0.02	11.07	12	0.52
D13505	0	12	10	11	6	2	9	0.56	0.01	26.26	24	0.34
Average	0	9.67	21.33	16.67	5.33	6.33	11.00					
R2												
K00821	0	0	3	0	0	0	0	0.50	0.02	2.92	3	0.40
AF083880	0	0	1	0	0	0	0	0.49	−0.01	0.91	2	0.34
AF124148	0	1	1	6	4	3	4	0.48	0.07	14.60	14	0.41
U13987	0	0	10	0	0	1	1	0.53	0.09*	5.23	5	0.39
E00532	0	0	0	1	0	0	0	0.77**	0.70**	17.95	1	0**
L10292	0	0	1	1	0	1	1	0.47	−0.03	2.12	3	0.55
AB030491	0	0	2	0	0	0	0	0.52	0.03	2.92	2	0.23
AF079058	0	1	0	0	0	0	0	0.57*	0.23*	5.18	1	0.02*
D31700	0	0	0	2	0	0	1	0.47	−0.07	2.92	3	0.40
J01298	0	0	3	2	0	2	2	0.58	0.07	9.00	5	0.11
AF089850	0	0	1	1	0	0	0	0.52	0.05	5.25	2	0.07
M94012	0	0	2	1	0	0	0	0.58**	0.21**	13.16	3	0.00**
AB004062	0	0	1	1	0	0	0	0.69**	0.28**	26.52	2	0***
M11317	0	0	3	3	0	0	0	0.49	0.01	6.00	6	0.42
Average	0	0.14	2.00	1.29	0.29	0.50	0.64					

R1 represents plant-environment interaction related reference genes, which include EU450800, the disease resistance gene *Rps1-k-1*; L20310, nodulin (nod-20) gene and D13505, early nodulin related gene; R2 signifies other fourteen reference genes.

C, Cultivars; W, Wild soybean.

Fixed, the number of fixed differences between cultivated and wild soybeans; In W, Mutations that are polymorphic in wild soybeans, but monomorphic in cultivars; In C, Mutations that are polymorphic in cultivars, but monomorphic in wild soybean; Shared, the total number of shared mutations.

*R*_M_, the minimum number of recombination events [25], both coding and noncoding sequences were considered.

* *P*<0.05,

** *P*<0.01,

*** *P*<0.001.

Three different genetic statistics (Chi-square statistic, *F_st_* and *S_nn_*) were employed to further examine genetic differentiation between wild and cultivated soybeans (Table 2). In our analysis, genetic differentiation was considered significant only when all of the three statistic test gave significant results (*P*<0.05). According to this criterion, none of these three genes showed significant genetic differentiation between cultivated and wild soybean. No significant genetic differentiation was detected for most of the reference genes expect for four genes, which showed significant genetic differentiation between cultivated and wild soybean. Collectively, both phylogenetic analysis and genetic differentiation tests showed no significant divergence between cultivated and wild soybeans for both the target and the reference genes, which could result from the short domestication time of soybean.

### Pseudogenes in the Two IFS Genes

The full *IFS1* alignment spanned 2,748bp including 64 sites with alignment gaps (indel polymorphisms). In the first exon, 13 replacement changes were identified. Among these replacement changes, there is a ‘T’ to ‘C’ mutation which causes the start codon, ‘ATG’, of *IFS1* gene of accession C_HC35 change to ‘ACG’. This may lead to no translation of *IFS1* gene in accession C_HC35 (Figure S4a).

The full *IFS2* alignment spanned 2,956bp including 24 sites with alignment gaps. In the first exon, four indels of 1 or 2bp were identified. These four indels were all singletons, two found in accession C_HC16, one in C_HC28 and one in C_HC31 (Figure S4b). These indels all caused frameshift mutations, leading to probable function loss of related genes.

One start codon mutation of *IFS1* in accession C_HC35 and a total of four frameshift mutations of *IFS2* gene in accession C_HC16, C_HC28 and C_HC31 may result in no function of the *IFS1* or *IFS2* in the corresponding accession plants. However, though IFS acts as the key metabolic entry point for the formation of all isoflavonoids [4], the silence of these genes showed no significant impact on isoflavone content of the corresponding accessions [14], indicating that these two IFS genes may be able to compensate for each other. Also, this was supported by previous study which showed both IFS1 and IFS2 enzyme could convert naringenin and liquiritigenin to genistein and daidzein respectively [6]. Due to such complementary effect of *IFS1* and *IFS2* genes, the harm caused by deleterious mutations can be overcome effectively.

### Different Selection Contexts of IFS Genes and F3H Gene

Tajima’s *D* was used to determine allele frequency changes by comparing the two *IFS* genes and the *F3H* gene with reference genes in cultivated and wild soybean. Interestingly, the results (Table 3) showed that *D* values at the three genes (−1.33 on average) in cultivars were slightly lower than that (−1.22 on average) in wild soybean, suggesting an excess of low-frequency nucleotide polymorphisms in both cultivated and wild soybean. However, *D* values at the fourteen R2 genes (0.03 on average) in cultivars were higher than that (−0.65 on average) in wild soybean, suggesting an excess of low-frequency nucleotide polymorphisms in wild soybean while more intermediate-frequency polymorphisms in cultivars, which was consistent with the theoretical expectation that some low-frequency variants have been preferentially lost in cultivars because of the recent bottleneck during domestication.

**Table 3:** Test for evolutionary forces shaping *IFS1*, *IFS2*, *F3H2* gene, only CDS were considered.

Gene	*Tajima’s D*		*K_a_*/*K_s_*	
	C	W	C	W
*IFS1*	−1.38	−2.20**	0.16	0.27
*IFS2*	−2.39***	−2.22**	0.25	0.56
*F3H2*	−0.21	0.76	1.19	0.81
Average	−1.33	−1.22		
R1				
EU450800	−1.39	−1.61	0.68	0.57
L20310	0.14	0.03	5.51	1.82
D13505	−1.28	−1.65	0.10	0.17
Average	−0.84	−1.07		
R2				
K00821	/	−1.38	0/0	0.33
AF083880	/	−1.16	0/0	1.53e−4*/*0
AF124148	0.96	−0.49	0.33	0.43
U13987	/	−1.19	0/0	0.34
E00532	−0.40	−0.49	6.93e−4/0	6.18e−4/0
L10292	0.24	−0.26	6.17e−4*/*0	0.46
AB030491	/	−1.07	0/0	0/0
AF079058	0.74	/	0.00	0/0
D31700	−1.49	−1.50	0.00	0.00
J01298	0.22	0.24	6.40e−4/5.07e−4	0.15
AF089850	0.85	−1.06	0.00	0.12
M94012	1.65	−0.40	1.39e−3/0	1.65e−3/0
AB004062	−0.45	1.61	5.26e−4/0	2.69e−3/0
M11317	1.02	−1.27	0.58	0.60
Average	0.33	−0.65		

R1 represents plant-environment interaction related reference genes, which include EU450800, the disease resistance gene *Rps1-k-1*; L20310, nodulin (nod-20) gene and D13505, early nodulin related gene; R2 signifies other fourteen reference genes.

C, Cultivars; W, Wild soybeans; All, Cultivars and Wild soybeans.

* 0.01<*P*<0.05;

** 0.001<*P*<0.01;

*** *P*<0.001.

The ratio of nonsynonymous (*Ka*) to synonymous (*Ks*) nucleotide substitutions, is widely used to evaluate selection effect. Under neutral evolution, there should be *Ka/Ks* = 1. In contrast, *Ka/Ks*<1 indicates a negative or purifying selection and *Ka/Ks*>1 on the other hand is a strong evidence showing positive or diversifying selection.

We calculated *Ka/Ks* ratio for the full length coding sequences of *IFS1*, *IFS2*, *F3H2* and the reference genes in cultivated and wild samples (Table 3). We excluded those loci with synonymous substitution rate <0.1%, because *Ks* (as denominator) was too small to get a reliable estimate. According to our results, no obvious positive selection was detected in the two *IFS* genes. However, for the *F3H2* gene in the cultivated accessions, *Ka/Ks*  = 1.19, which suggested positive selection or relaxation of selection pressure during and after domestication process. Interestingly, similar selection pattern was found in the three R1 genes. At one of these three R1 genes (L20310), there was significantly positive selection detected in both cultivated (*Ka/Ks*  = 5.51) and wild (*Ka/Ks*  = 1.82) soybeans. While in the fourteen R2 genes, there was no positive selection detected in both cultivated and wild soybeans.

## Discussion

### Unique Evolution Scenario of IFS Genes and F3H2 Gene During Soybean Domestication

Our results reveal two remarkable phenomena: consistent polymorphism pattern between the target genes and three plant-environment interaction related reference genes (R1), contrastive evolutionary characteristics between environmental response related genes and other functional genes (R2). Previous studies have indicated that these three targeted genes play important roles in soybean’s resistance to diverse environmental stresses [4]. As well as, three reference genes are involved in pathogens or rhizobiums interaction. Therefore, similar functional genes might share unique evolutionary patterns, e.g. shared evolutionary parameters were detected in these genes, including high nucleotide diversity, frequent sequence exchange and prone to positive selection. These environmental response related genes face a variety of biotic or abiotic factors, making them co-evolve with pathogens or other stress [15]. On the contrary, other reference genes appear critical to soybean’s growth and some of them are connected with important agricultural characters of domesticated soybean. Hence, they are more conservative in nucleotide sequences.

In addition, inconsistent evolutionary signatures between environmental response related genes and R2 were detected in cultivated and wild soybeans. For example, much higher nucleotide diversity (θ), more frequent recombination and more negative Tajima’s D values were detected in wild soybeans than those of cultivated soybeans for R2 genes. In the environmental response related genes, however, shared level of those values between cultivated and wild soybeans were detected. Considering the environmental stress faced by the environmental response related genes, either biotic or abiotic, may have not changed much for either wild or cultivated soybean, the high level of nucleotide diversity therefore may be important to such functional requirements for both, especially considering many polymorphism sites which shared between cultivated and wild soybean (Table 2). The nucleotide diversity (θ) at these environmental response related genes showed no significant difference (*t*-test, *P* = 0.30) between the cultivated and wild ones. This disparity is inconsistent with the commonly believed diversity loss after domestication. There must be some forces that can contribute to the quick recovery of diversity in these three target genes, for example intentional or accidental gene introgression during soybean domestication. In contrast, for R2 genes, the nucleotide diversity (θ) in cultivated soybeans were significantly (*t*-test, *P*<0.01) lower than those in the wild ones. These fourteen reference genes appear critical to soybean’s growth and some of them are connected with important agricultural characters of domesticated soybean. Therefore, both bottleneck effect and probable artificial selection may play important role in the reduction of their nucleotide diversity in cultivated soybean relative to wild soybean [3].

The allele frequency changes determined by Tajima’s *D* showed similar conclusion. The Tajima’s D values detected in the environmental response related genes were similarly minus in both two populations (Table 3), indicating that the mutations in these genes were mostly low-frequency changes which had vague relationship with population structure. As for the reference genes, however, the Tajima’s D values were much higher in the cultivated than the wild soybean, suggesting much higher mutation frequency in the reference genes of the cultivated soybean, which could be the results of the removal of the rare alleles in the process of artificial selection.

Based on above observations, the evolution scenario of these three target genes can be inferred as follows: for *IFS1*, *IFS2* and especially *F3H2* genes, frequent gene introgression and recombination may have occurred in the domestication history of soybean, introducing considerable amount of diversities. And these relative large diversities are kept either simply by hitchhiking effects or due to the unique functional requirements of these three genes.

### F3H2 Gene Evolves Faster than the Two IFS Genes

According to our results, we found that *F3H2* gene enjoys a faster evolution rate than those two *IFS* genes based on the observation that the nucleotide diversity level of *F3H2* gene is higher than that of *IFS1* and *IFS2*, both in wild and cultivated soybean populations. Understanding the heterogeneity of evolutionary rate would shed light on the origin of large number of polymorphisms.

The recombination events found in *F3H2* gene is more frequent than those of the two *IFS* genes, either in wild and cultivated soybean populations, suggesting frequent sequence exchange between homologs was an important mechanism for generating diversity. The frequency of recombination between two homologs was commonly negatively correlated with distance and positively correlated with sequence similarity [16]. The higher nucleotide similarity and closer distance between *F3H1* and *F3H2* resulted in more frequent recombination than *IFS1* and *IFS2*, which in turn cause the higher level of diversity in *F3H2* than *IFS* genes. And this point is further confirmed in the linked polymorphism sites and phylogenetic analysis, in which we observed a group of *F3H2* genes are mixed with *F3H1* gene.

Furthermore, positive selection was observed in *F3H2* gene (Table 3), especially in the cultivated population, but not detected in *IFS1* and *IFS2* (Table 3), which shows a considerable diversifying selection force driving the evolution of *F3H2* gene. Theoretically, certain types of mutations are preferentially preserved for specific characters in adaption. Therefore, such diversifying selection of *F3H2* gene may reflect its functional importance in soybean’s adaption to the environment. As introduced above, *IFS* genes serve as the entry point of isoflavonoids biosynthesis pathway in legume plants and some other non-legume crops. According to previous study, these two genes are very conservative even between distant species. For instance, both *IFS* genes encode proteins in sugarbeet show a surprisingly >95% similarity to soybean IFS1 protein [6]. In contrast, *F3H* gene, which plays a major role in biosynthesis of flavonoids, seems to enjoy a different context of evolution. Previous studies indicated that flavonoids have key roles in diverse functions, a major part of which relates to providing defense to various microbes and environmental stress, such as UV light etc. Our previous association analysis showed that some SNPs in *F3H* were associated with SMV resistance [13]. The SMV response association analysis in our study revealed that both resistance and susceptible reactions composite in most clearly separate genetic groups, implying long-time coexistence of these phenotypes. One explanation for the result was that this defense function is critical for plants to live under various biotic and abiotic stresses in nature, but may also have considerable fitness costs as resistance genes (R genes) in plants. Therefore, balancing selection may operate on plant *F3H* genes and maintain different phenotypes in a frequency-dependent manner for a long time.

## Materials and Methods

### Plant Materials

The seeds of 33 Chinese soybean accessions consisted of 16 wild and 17 cultivated accessions were provided by Germplasm Storage of Chinese National Center for Soybean Improvement (Nanjing Agricultural University, Nanjing, China). These accessions were distributed in 19–49°N and 106–131°E. This sampling strategy was designed to not only cover all the six soybean ecological habitats in China [17] but also take into account the broad spectrum of different levels of seed isoflavone and flavonoids concentration in Chinese soybeans. Plants were grown at Jiangpu Experimental Station of Nanjing Agricultural University (Nanjing, China). The plant materials were listed in Online Resource (Table S1).

### Selection of Reference Gene Loci

Based on the facts that *IFS* and *F3H* genes are involved in stress response, we chose three genes interacting with microbes, for example EU450800, the disease resistance gene *Rps1-k-1*; L20310, nodulin (nod-20) gene and D13505, early nodulin related gene, grouping as R1. Meanwhile, fourteen other genes were randomly selected to represent a range of functions [3], classified as R2 (Table S2). Sequences of reference genes were obtained from 31 re-sequencing wild and cultivated soybean genomes [18]. To test whether there is a genetic differentiation between 33 accessions in our study and the 31 re-sequencing ones, four reference genes were sequenced in 33 accessions used in this study and compared with the sequences from the re-sequencing database. The four reference genes include MAT9 gene (M94012), A5A4B3 glycinin gene (AB004062), urate-degrading peroxidase gene (AF089850) and low molecular weight heat shock protein gene (M11317). Three types of statistical tests, (Chi-square statistic, *F*_st_ and *S*_nn_, as described in “Analysis of genetic structure”) were applied to detect genetic differentiation between accessions used in our study and the ones from re-sequencing project. The results demonstrated that there was no significant genetic difference between the two populations for the four loci tested (Table S3).

### Cloning and Sequencing

Total genomic DNA was extracted from bulk leaf tissue of 8–10 *G. soja* or *G. max* plants as described by Keim et al. [19]. For the two *IFS* genes, we obtained partial promoter region, 5′-UTR, complete CDS, all introns and partial 3′-UTR. For the *F3H2* gene, most CDS and all introns were obtained. For the four reference genes, we used the primers published by Hyten et al. [3]. The primers were listed in Table S4. The process of PCR, cloning and sequencing were described in our previous study [13], [14]. Sequences of *IFS* and *F3H* genes were deposited in GenBank under accession number from EU391427 to EU391525. Sequences of the four reference genes have also been deposited in GenBank accession number JQ660375-JQ660504.

### Sequence Analysis

The sequencing results were assembled using BioLign software (http://en.bio-soft.net/dna/BioLign.html) and aligned by ClustalX software version 1.83 by manual check [20]. The nucleotide alignments were analyzed using DnaSP version 5.0 [21]. Indels were excluded from all estimates. Haplotype diversity (*H_d_*) was calculated using Equation 8.4 [22], except that *n* was used instead of 2*n*. Nucleotide diversity was estimated by π with Jukes and Cantor correction [23] and by θ from the number of polymorphic segregating (S) sites [24]. The divergences between species were estimated by *D*_xy_ and divergence at silent sites (*K_sil_*) with the Jukes and Cantor correction [22]. The minimum numbers of recombination events (*R*_M_) were estimated [25]. Phylogenetic trees were constructed based on the bootstrap neighbor-joining (NJ) method with a Kimura 2-parameter model by MEGA version 4.0 [26]. Coding sequences (CDSs) of *F3H1* (Glyma02g05450.1) alleles were download and employed as outgroup for soybean *F3H2* phylogenetic study (GenBank accession numbers: AY669324, AY669325, AY595420, AY994154, BT096691 and FJ770474)**.** The stability of internal nodes was assessed by bootstrap analysis with 1,000 replicates.

DnaSP was also used to perform a statistical test of neutrality, Tajima’s D test [27]. In order to evaluate the power of selection, we estimated the ratio of nonsynonymous (*Ka*) to synonymous (*Ks*) nucleotide substitutions (*Ka/Ks*) according to Nei and Gojobori’s equations [28]. Protein sequences were initially aligned by ClustalX [20], and the resulting amino acid sequence alignments were then used to guide the alignments of nucleotide CDSs using MEGA version 4.0.

### Analysis of Genetic Structure

Two distinct classes of test, haplotype-based statistical test (Chi-square statistic) and sequence-based statistical test (*F*_st_ and *S*_nn_), were applied to detect genetic differentiation between wild and cultivated soybean accessions. The *F*_st_ statistic [29] was calculated by ARLEQUIN version 3.11 software with permuting the data 1,000 times [30]. The nearest-neighbors statistic (*S*_nn_) measures how often the “nearest-neighbors” (in sequence space) of sequences and appears to be the most powerful statistic or nearly as powerful as the best statistic method under all conditions examined [31]. The statistical significance of pairwise *S*_nn_ values was determined by permuting the data 1000 times in DnaSP v 5.0. The genetic differentiation were also estimated by using *χ* ^2^ test [22], which can be directly adapted to use with nucleotide variation by treating each distinct haplotypes as an allele.

## Supporting Information

Figure S1 **Biosynthesis of isoflavonoids and flavonoids in soybean.** F3H, flavonone-3-hydroxylase; FLS, Flavonol synthase; FNS, Flavone synthase; IFS, isoflavanone synthase. Hollowed arrows represent multiple or uncertain steps.
(DOC)

Figure S2 **Nucleotide polymorphism sites of** ***IFS1*** **(a) and** ***IFS2*** **(b) genes.** The locations of the polymorphism sites were shown in the above line. The polymorphic sites were highlighted by red color.
(DOC)

Figure S3 **Phylogenetic trees of the seventeen reference genes constructed by neighbor-joining (NJ) method.**
(PDF)

Figure S4 **(a) Patial (from start codon to 121bp) alignment of** ***IFS1*** **coding sequence, start codon mutant of accession C_HC35 was shown (red line).** (b) Patial aliagnment of *IFS2* coding sequence, four indels of 1 or 2bp, two found in accession C_HC16, one in C_HC28 and one in C_HC31, were shown (red arrow).
(DOC)

Table S1 **Thirty-three soybean accessions included in this study.**
(DOC)

Table S2 **GenBank accession numbers, functional description and Gene Ontology for reference genes.**
(DOC)

Table S3 **Genetic differentiation between soybean accessions in our study and the re-sequencing population.**
(DOC)

Table S4 **Primer information for** ***IFS1*** **,** ***IFS2*** **and** ***F3H2*** **gene and four reference genes.**
(DOC)

Table S5 **Nucleotide diversity (%) within and between groups at** ***F3H2.***
(DOC)
