# Supplementary material for: Diversifying Selection on Flavanone 3-Hydroxylase and Isoflavone Synthase Genes in Cultivated Soybean and Its Wild Progenitors
Source: PLoS One. 2013 Jan 16;8(1):e54154. doi: 10.1371/journal.pone.0054154 (PMC3546919; doi:10.1371/journal.pone.0054154)

**Figure S1.** Biosynthesis of isoflavonoids and flavonoids in soybean. F3H, flavonone-3-hydroxylase; FLS, Flavonol synthase; FNS, Flavone synthase; IFS, isoflavanone synthase. Hollowed arrows represent multiple or uncertain steps.


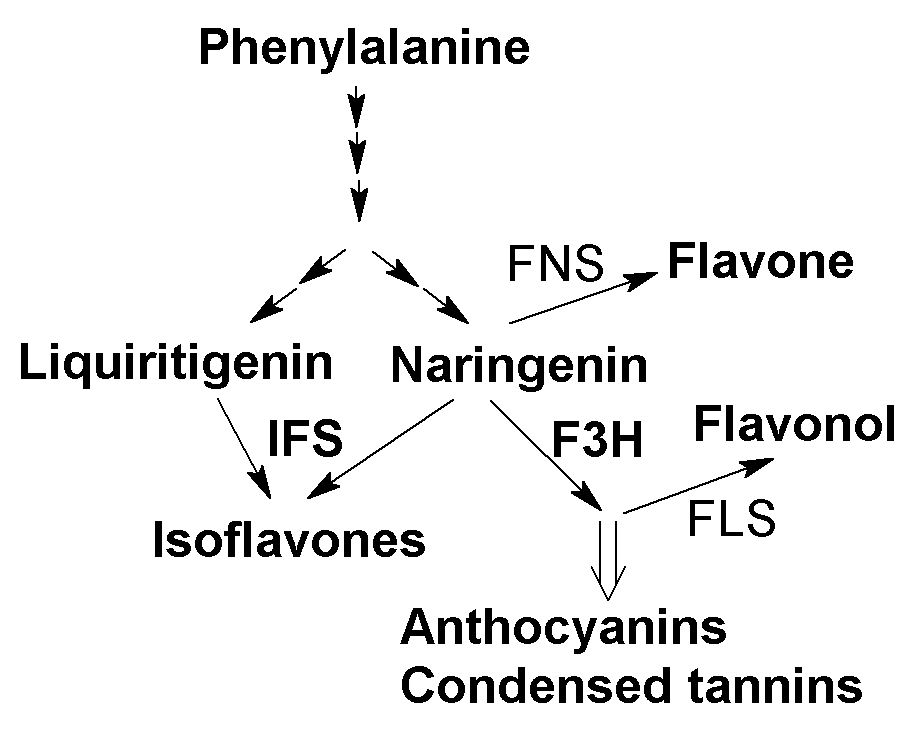

Supplement: Figure S1 — Biosynthesis of isoflavonoids and flavonoids in soybean. F3H, flavonone-3-hydroxylase; FLS, Flavonol synthase; FNS, Flavone synthase; IFS, isoflavanone synthase. Hollowed arrows represent multiple or uncertain steps. (DOC) [file pone.0054154.s001.doc]
