# Supplementary material for: Diversifying Selection on Flavanone 3-Hydroxylase and Isoflavone Synthase Genes in Cultivated Soybean and Its Wild Progenitors
Source: PLoS One. 2013 Jan 16;8(1):e54154. doi: 10.1371/journal.pone.0054154 (PMC3546919; doi:10.1371/journal.pone.0054154)

**Figure S2 Nucleotide polymorphism sites of *IFS1* (a) and *IFS2* (b) genes**

The locations of the polymorphism sites were shown in the above line. The polymorphic sites were highlighted by red color.


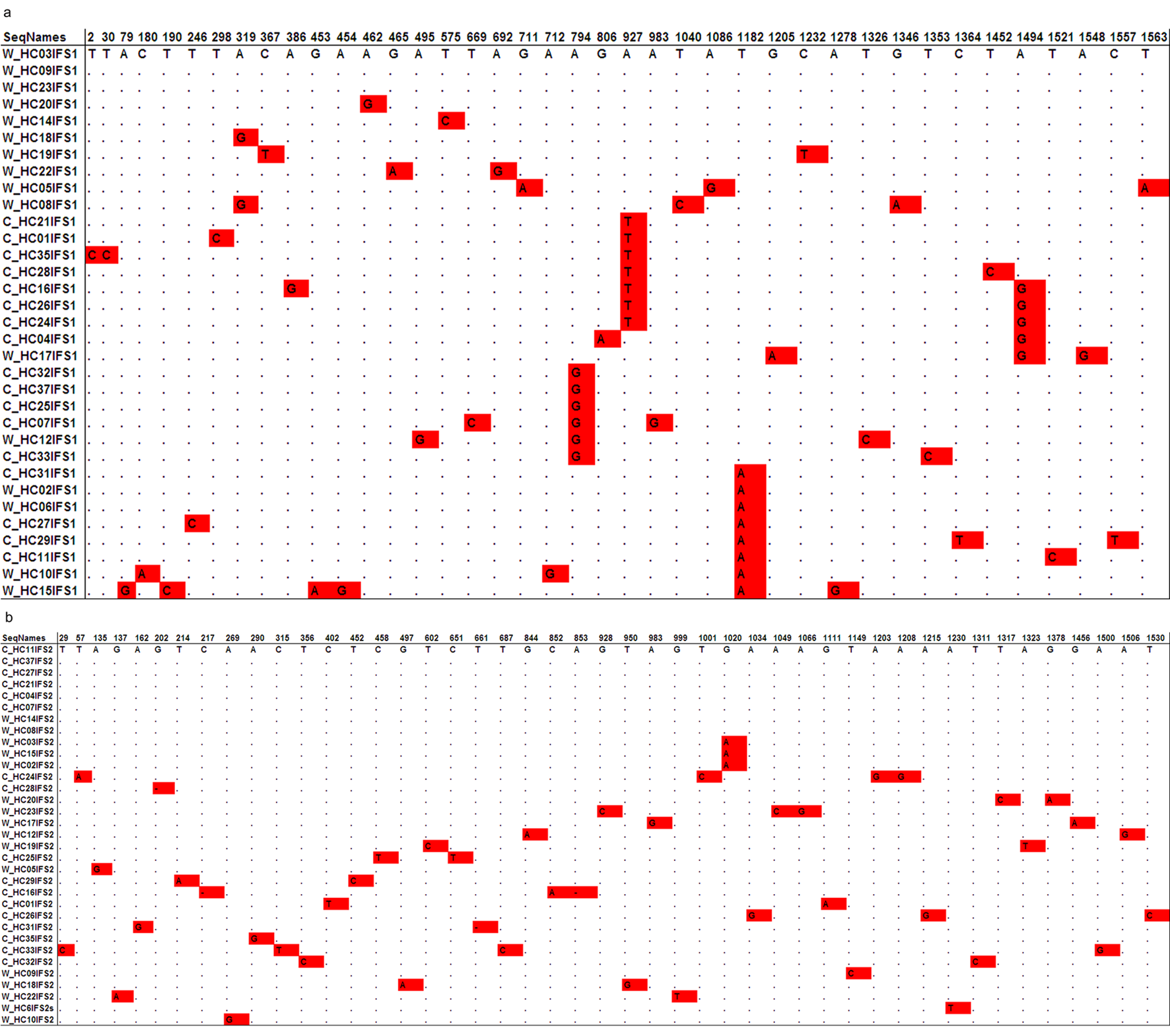

Supplement: Figure S2 — Nucleotide polymorphism sites of IFS1 (a) and IFS2 (b) genes. The locations of the polymorphism sites were shown in the above line. The polymorphic sites were highlighted by red color. (DOC) [file pone.0054154.s002.doc]
