# Supplementary material for: Diversifying Selection on Flavanone 3-Hydroxylase and Isoflavone Synthase Genes in Cultivated Soybean and Its Wild Progenitors
Source: PLoS One. 2013 Jan 16;8(1):e54154. doi: 10.1371/journal.pone.0054154 (PMC3546919; doi:10.1371/journal.pone.0054154)
Supplement: Figure S3 — Phylogenetic trees of the seventeen reference genes constructed by neighbor-joining (NJ) method. (PDF) [file pone.0054154.s003.pdf]

Figure S3.

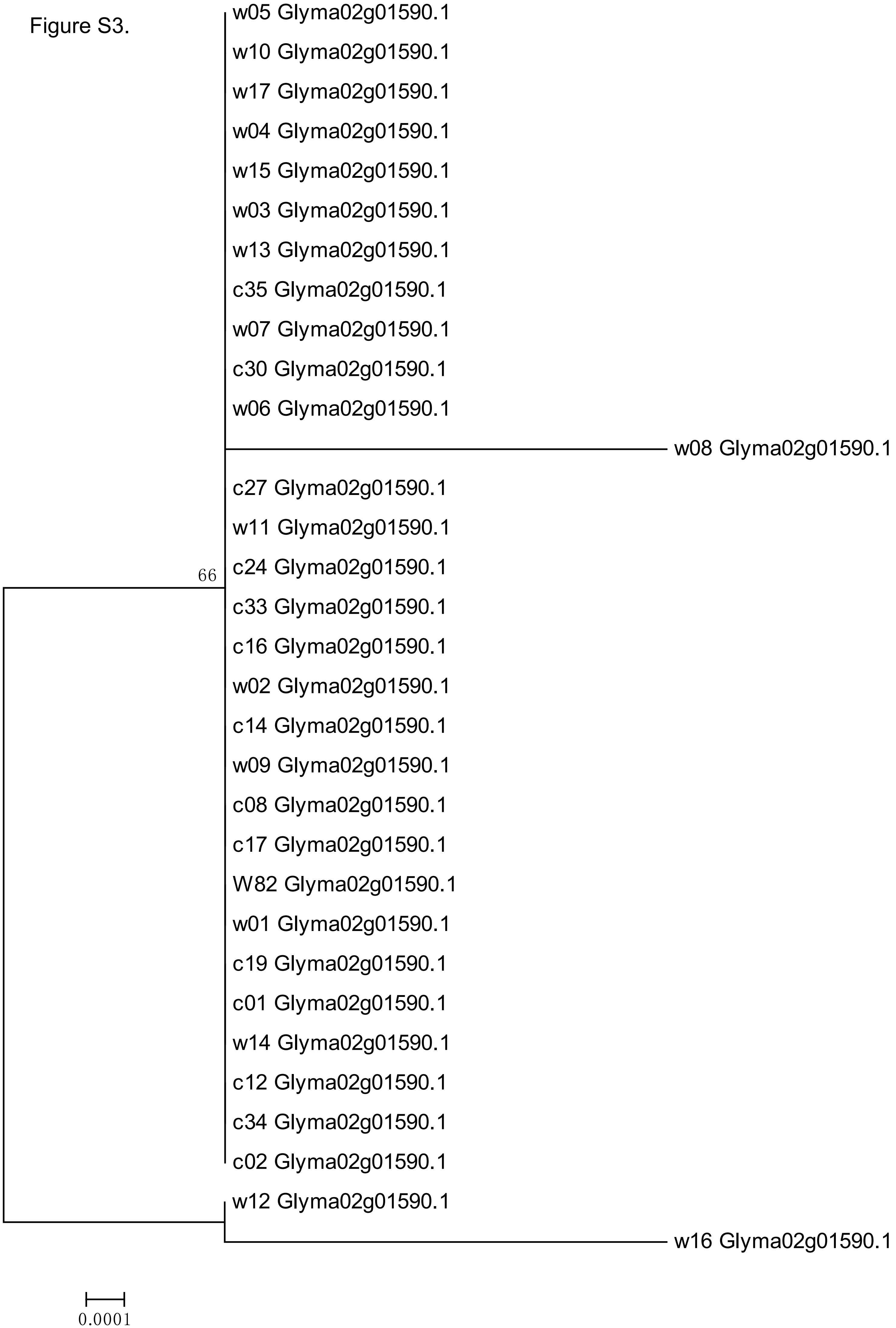

w05 Glyma02g01590.1
w10 Glyma02g01590.1
w17 Glyma02g01590.1
w04 Glyma02g01590.1
w15 Glyma02g01590.1
w03 Glyma02g01590.1
w13 Glyma02g01590.1
c35 Glyma02g01590.1
w07 Glyma02g01590.1
c30 Glyma02g01590.1
w06 Glyma02g01590.1
w08 Glyma02g01590.1
c27 Glyma02g01590.1
w11 Glyma02g01590.1
66
c24 Glyma02g01590.1
c33 Glyma02g01590.1
c16 Glyma02g01590.1
w02 Glyma02g01590.1
c14 Glyma02g01590.1
w09 Glyma02g01590.1
c08 Glyma02g01590.1
c17 Glyma02g01590.1
W82 Glyma02g01590.1
w01 Glyma02g01590.1
c19 Glyma02g01590.1
c01 Glyma02g01590.1
w14 Glyma02g01590.1
c12 Glyma02g01590.1
c34 Glyma02g01590.1
c02 Glyma02g01590.1
w12 Glyma02g01590.1
w16 Glyma02g01590.1
0.0001

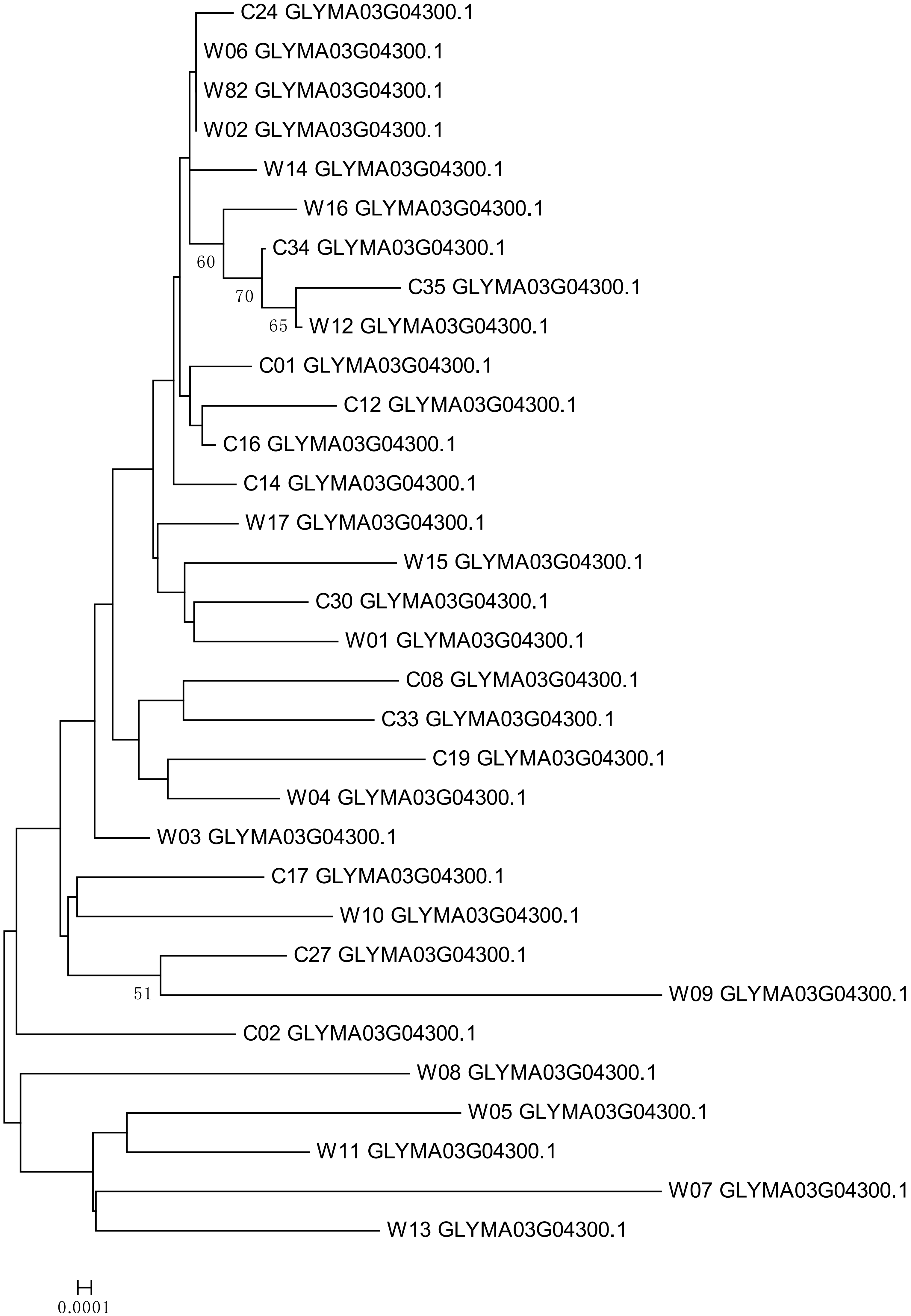
C24 GLYMA03G04300.1
W06 GLYMA03G04300.1
W82 GLYMA03G04300.1
W02 GLYMA03G04300.1
W14 GLYMA03G04300.1
W16 GLYMA03G04300.1
60
C34 GLYMA03G04300.1
70
C35 GLYMA03G04300.1
65
W12 GLYMA03G04300.1
C01 GLYMA03G04300.1
C12 GLYMA03G04300.1
C16 GLYMA03G04300.1
C14 GLYMA03G04300.1
W17 GLYMA03G04300.1
W15 GLYMA03G04300.1
C30 GLYMA03G04300.1
W01 GLYMA03G04300.1
C08 GLYMA03G04300.1
C33 GLYMA03G04300.1
C19 GLYMA03G04300.1
W04 GLYMA03G04300.1
W03 GLYMA03G04300.1
C17 GLYMA03G04300.1
W10 GLYMA03G04300.1
C27 GLYMA03G04300.1
51
W09 GLYMA03G04300.1
C02 GLYMA03G04300.1
W08 GLYMA03G04300.1
W05 GLYMA03G04300.1
W11 GLYMA03G04300.1
W07 GLYMA03G04300.1
W13 GLYMA03G04300.1
0.0001

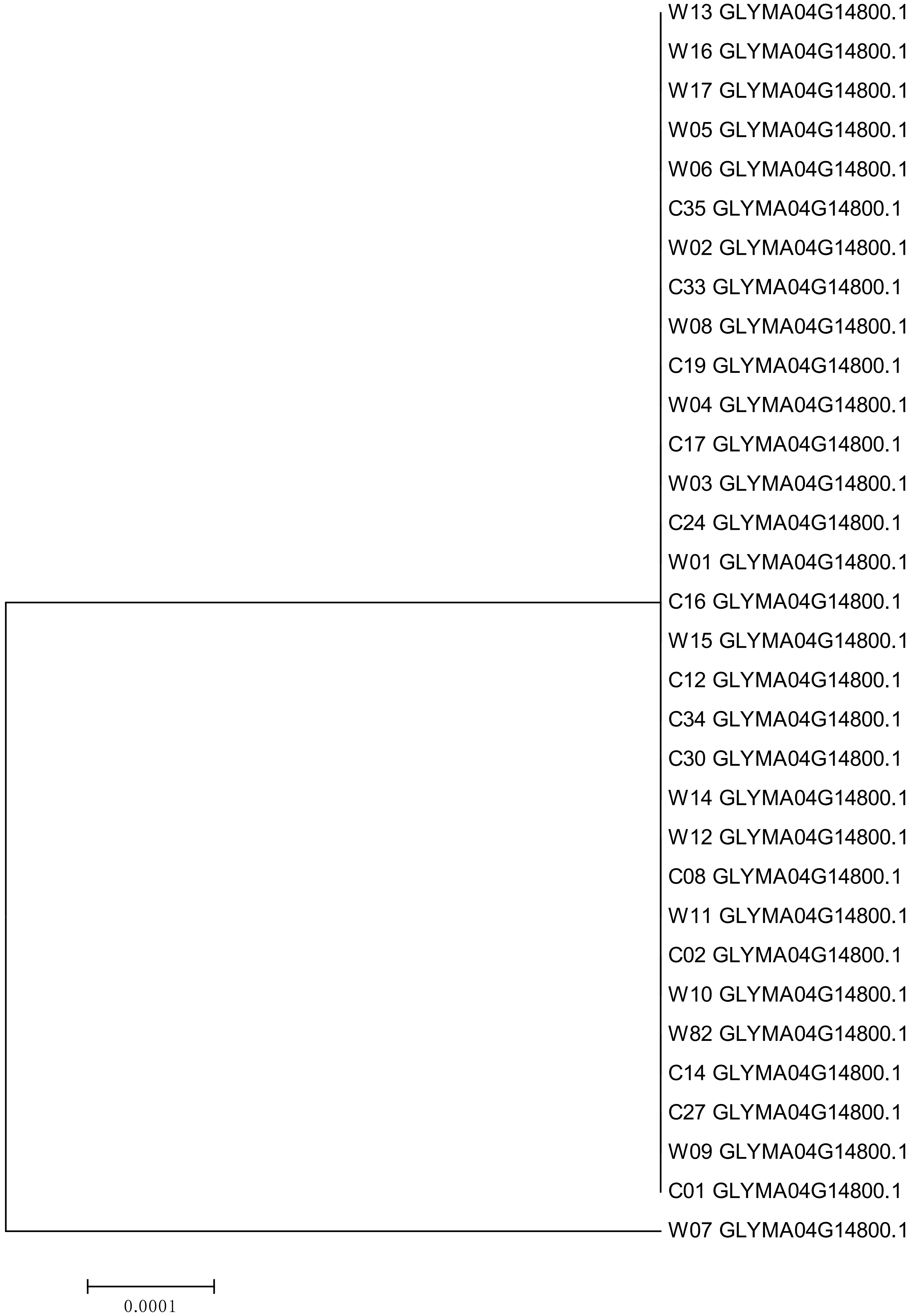
W13 GLYMA04G14800.1
W16 GLYMA04G14800.1
W17 GLYMA04G14800.1
W05 GLYMA04G14800.1
W06 GLYMA04G14800.1
C35 GLYMA04G14800.1
W02 GLYMA04G14800.1
C33 GLYMA04G14800.1
W08 GLYMA04G14800.1
C19 GLYMA04G14800.1
W04 GLYMA04G14800.1
C17 GLYMA04G14800.1
W03 GLYMA04G14800.1
C24 GLYMA04G14800.1
W01 GLYMA04G14800.1
C16 GLYMA04G14800.1
W15 GLYMA04G14800.1
C12 GLYMA04G14800.1
C34 GLYMA04G14800.1
C30 GLYMA04G14800.1
W14 GLYMA04G14800.1
W12 GLYMA04G14800.1
C08 GLYMA04G14800.1
W11 GLYMA04G14800.1
C02 GLYMA04G14800.1
W10 GLYMA04G14800.1
W82 GLYMA04G14800.1
C14 GLYMA04G14800.1
C27 GLYMA04G14800.1
W09 GLYMA04G14800.1
C01 GLYMA04G14800.1
W07 GLYMA04G14800.1
0.0001

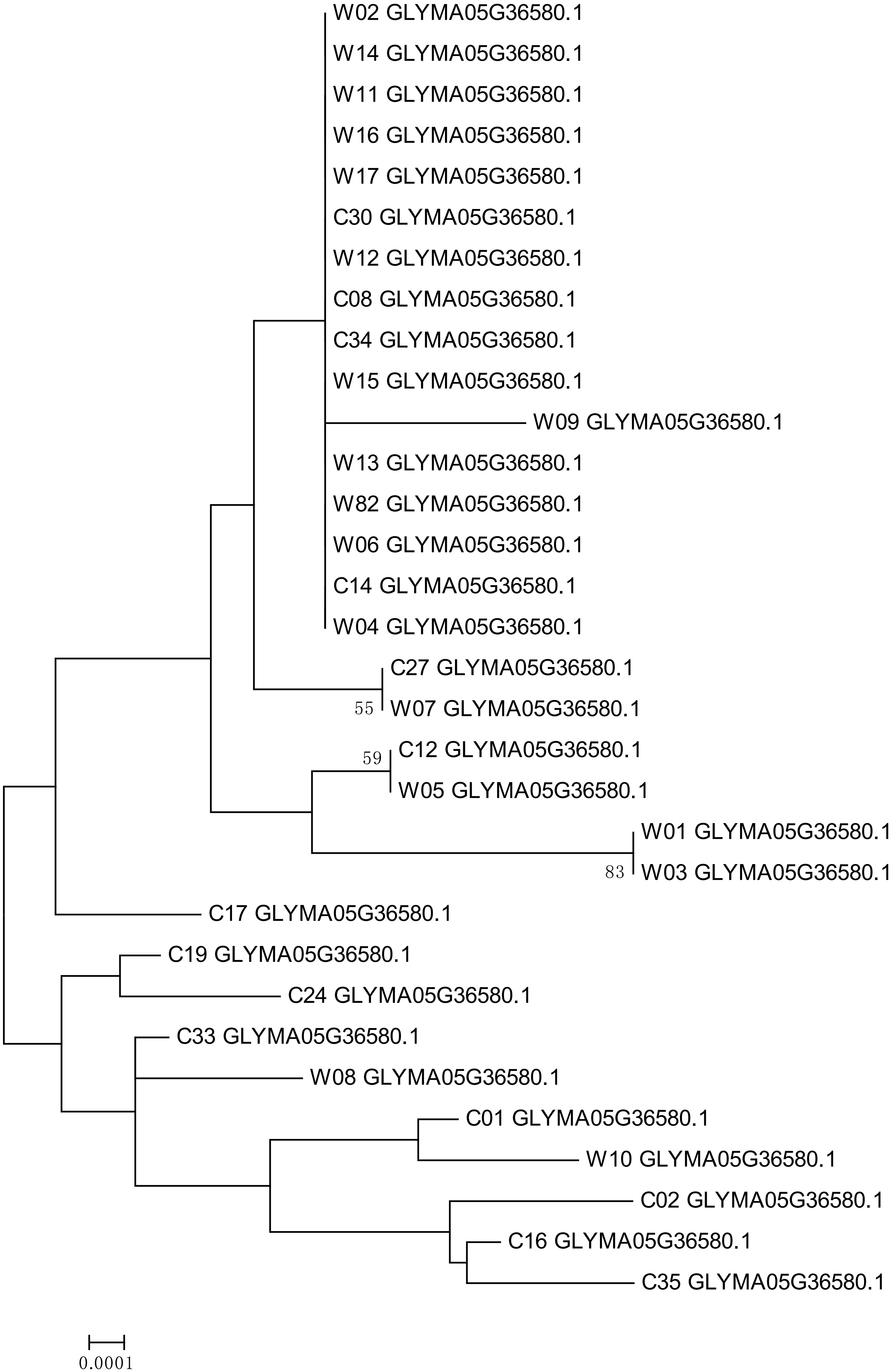
W02 GLYMA05G36580.1
W14 GLYMA05G36580.1
W11 GLYMA05G36580.1
W16 GLYMA05G36580.1
W17 GLYMA05G36580.1
C30 GLYMA05G36580.1
W12 GLYMA05G36580.1
C08 GLYMA05G36580.1
C34 GLYMA05G36580.1
W15 GLYMA05G36580.1
W09 GLYMA05G36580.1
W13 GLYMA05G36580.1
W82 GLYMA05G36580.1
W06 GLYMA05G36580.1
C14 GLYMA05G36580.1
W04 GLYMA05G36580.1
C27 GLYMA05G36580.1
55
W07 GLYMA05G36580.1
59
C12 GLYMA05G36580.1
W05 GLYMA05G36580.1
W01 GLYMA05G36580.1
83
W03 GLYMA05G36580.1
C17 GLYMA05G36580.1
C19 GLYMA05G36580.1
C24 GLYMA05G36580.1
C33 GLYMA05G36580.1
W08 GLYMA05G36580.1
C01 GLYMA05G36580.1
W10 GLYMA05G36580.1
C02 GLYMA05G36580.1
C16 GLYMA05G36580.1
C35 GLYMA05G36580.1
0.0001

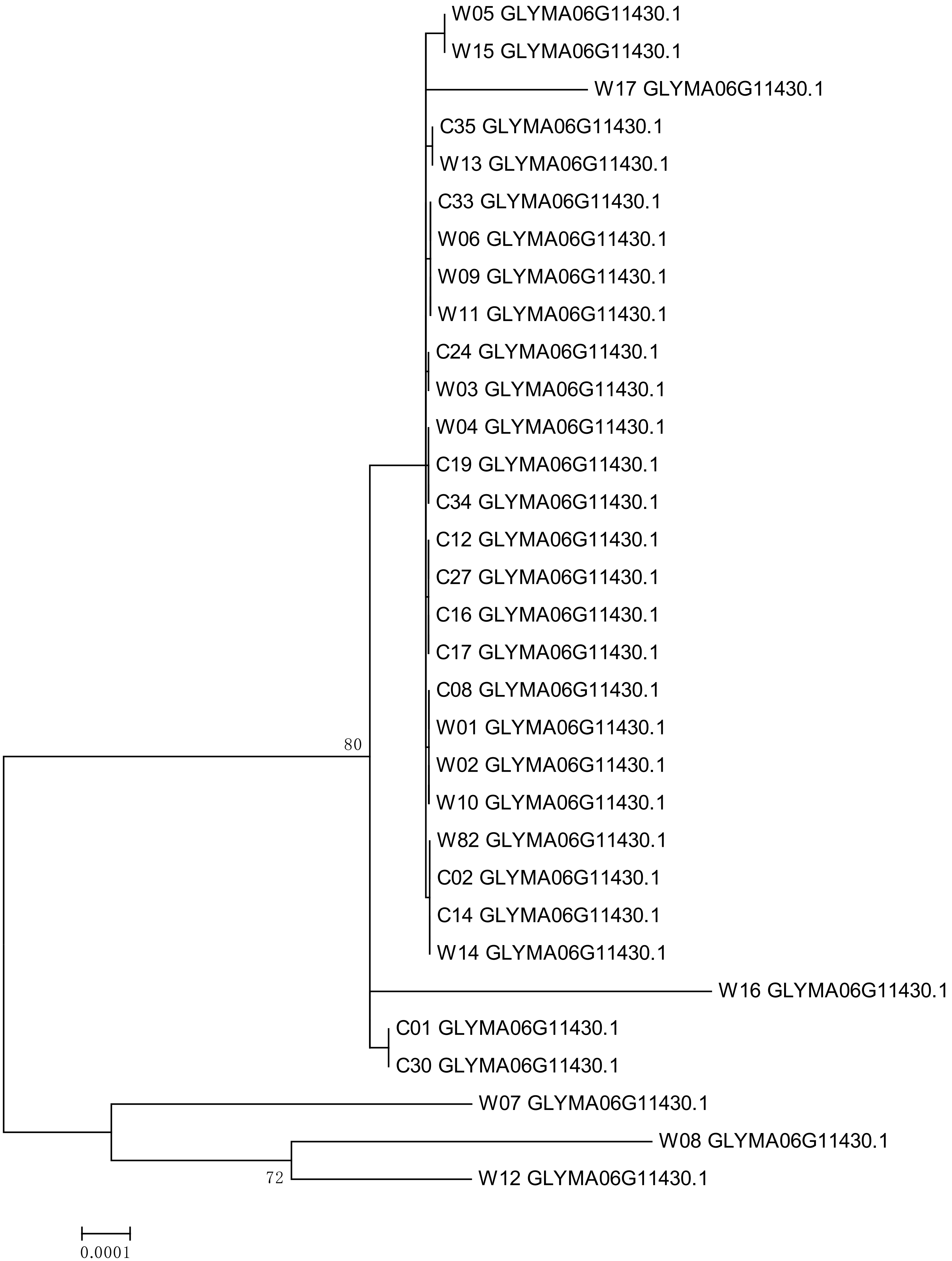
W05 GLYMA06G11430.1
W15 GLYMA06G11430.1
W17 GLYMA06G11430.1
C35 GLYMA06G11430.1
W13 GLYMA06G11430.1
C33 GLYMA06G11430.1
W06 GLYMA06G11430.1
W09 GLYMA06G11430.1
W11 GLYMA06G11430.1
C24 GLYMA06G11430.1
W03 GLYMA06G11430.1
W04 GLYMA06G11430.1
C19 GLYMA06G11430.1
C34 GLYMA06G11430.1
C12 GLYMA06G11430.1
C27 GLYMA06G11430.1
C16 GLYMA06G11430.1
C17 GLYMA06G11430.1
C08 GLYMA06G11430.1
W01 GLYMA06G11430.1
W02 GLYMA06G11430.1
W10 GLYMA06G11430.1
W82 GLYMA06G11430.1
C02 GLYMA06G11430.1
C14 GLYMA06G11430.1
W14 GLYMA06G11430.1
80
W16 GLYMA06G11430.1
C01 GLYMA06G11430.1
C30 GLYMA06G11430.1
W07 GLYMA06G11430.1
W08 GLYMA06G11430.1
72
W12 GLYMA06G11430.1
0.0001

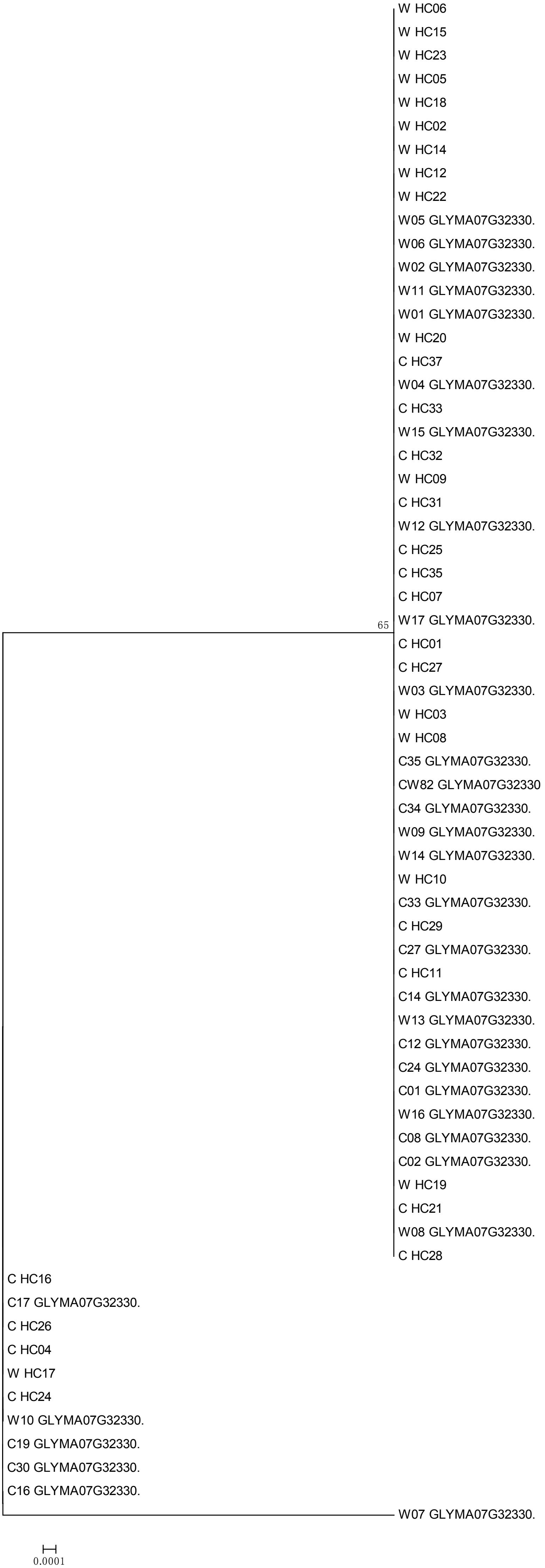
W HC06
W HC15
W HC23
W HC05
W HC18
W HC02
W HC14
W HC12
W HC22
W05 GLYMA07G32330.
W06 GLYMA07G32330.
W02 GLYMA07G32330.
W11 GLYMA07G32330.
W01 GLYMA07G32330.
W HC20
C HC37
W04 GLYMA07G32330.
C HC33
W15 GLYMA07G32330.
C HC32
W HC09
C HC31
W12 GLYMA07G32330.
C HC25
C HC35
C HC07
65
W17 GLYMA07G32330.
C HC01
C HC27
W03 GLYMA07G32330.
W HC03
W HC08
C35 GLYMA07G32330.
CW82 GLYMA07G32330
C34 GLYMA07G32330.
W09 GLYMA07G32330.
W14 GLYMA07G32330.
W HC10
C33 GLYMA07G32330.
C HC29
C27 GLYMA07G32330.
C HC11
C14 GLYMA07G32330.
W13 GLYMA07G32330.
C12 GLYMA07G32330.
C24 GLYMA07G32330.
C01 GLYMA07G32330.
W16 GLYMA07G32330.
C08 GLYMA07G32330.
C02 GLYMA07G32330.
W HC19
C HC21
W08 GLYMA07G32330.
C HC28
C HC16
C17 GLYMA07G32330.
C HC26
C HC04
W HC17
C HC24
W10 GLYMA07G32330.
C19 GLYMA07G32330.
C30 GLYMA07G32330.
C16 GLYMA07G32330.
W07 GLYMA07G32330.
0.0001

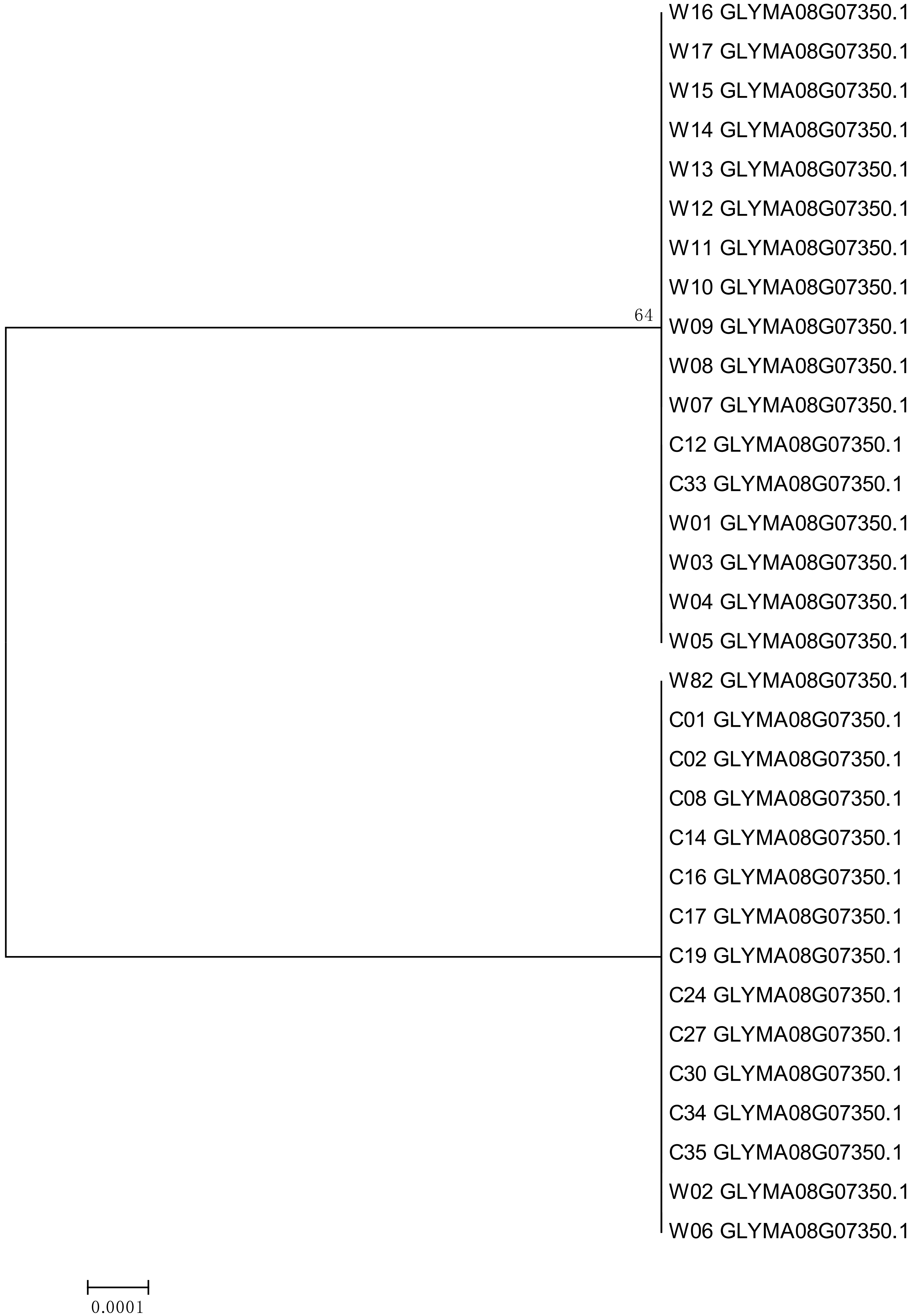
W16 GLYMA08G07350.1
W17 GLYMA08G07350.1
W15 GLYMA08G07350.1
W14 GLYMA08G07350.1
W13 GLYMA08G07350.1
W12 GLYMA08G07350.1
W11 GLYMA08G07350.1
W10 GLYMA08G07350.1
64
W09 GLYMA08G07350.1
W08 GLYMA08G07350.1
W07 GLYMA08G07350.1
C12 GLYMA08G07350.1
C33 GLYMA08G07350.1
W01 GLYMA08G07350.1
W03 GLYMA08G07350.1
W04 GLYMA08G07350.1
W05 GLYMA08G07350.1
W82 GLYMA08G07350.1
C01 GLYMA08G07350.1
C02 GLYMA08G07350.1
C08 GLYMA08G07350.1
C14 GLYMA08G07350.1
C16 GLYMA08G07350.1
C17 GLYMA08G07350.1
C19 GLYMA08G07350.1
C24 GLYMA08G07350.1
C27 GLYMA08G07350.1
C30 GLYMA08G07350.1
C34 GLYMA08G07350.1
C35 GLYMA08G07350.1
W02 GLYMA08G07350.1
W06 GLYMA08G07350.1
0.0001

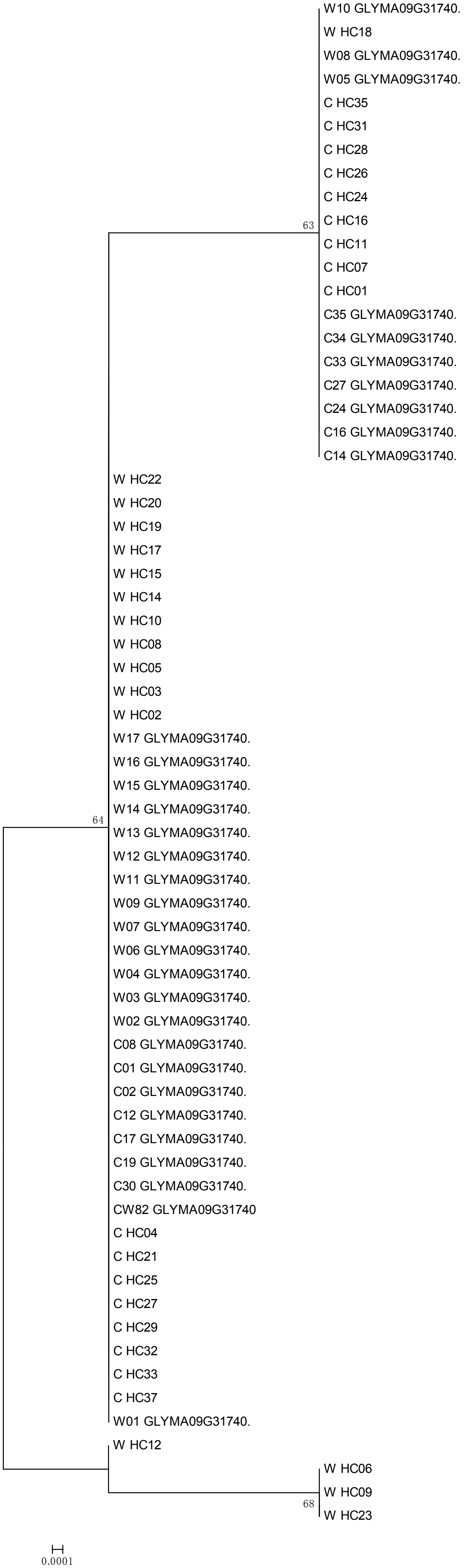
W10 GLYMA09G31740.
W HC18
W08 GLYMA09G31740.
W05 GLYMA09G31740.
C HC35
C HC31
C HC28
C HC26
C HC24
63
C HC16
C HC11
C HC07
C HC01
C35 GLYMA09G31740.
C34 GLYMA09G31740.
C33 GLYMA09G31740.
C27 GLYMA09G31740.
C24 GLYMA09G31740.
C16 GLYMA09G31740.
C14 GLYMA09G31740.
W HC22
W HC20
W HC19
W HC17
W HC15
W HC14
W HC10
W HC08
W HC05
W HC03
W HC02
W17 GLYMA09G31740.
W16 GLYMA09G31740.
W15 GLYMA09G31740.
W14 GLYMA09G31740.
64
W13 GLYMA09G31740.
W12 GLYMA09G31740.
W11 GLYMA09G31740.
W09 GLYMA09G31740.
W07 GLYMA09G31740.
W06 GLYMA09G31740.
W04 GLYMA09G31740.
W03 GLYMA09G31740.
W02 GLYMA09G31740.
C08 GLYMA09G31740.
C01 GLYMA09G31740.
C02 GLYMA09G31740.
C12 GLYMA09G31740.
C17 GLYMA09G31740.
C19 GLYMA09G31740.
C30 GLYMA09G31740.
CW82 GLYMA09G31740
C HC04
C HC21
C HC25
C HC27
C HC29
C HC32
C HC33
C HC37
W01 GLYMA09G31740.
W HC12
W HC06
W HC09
68
W HC23
0.0001

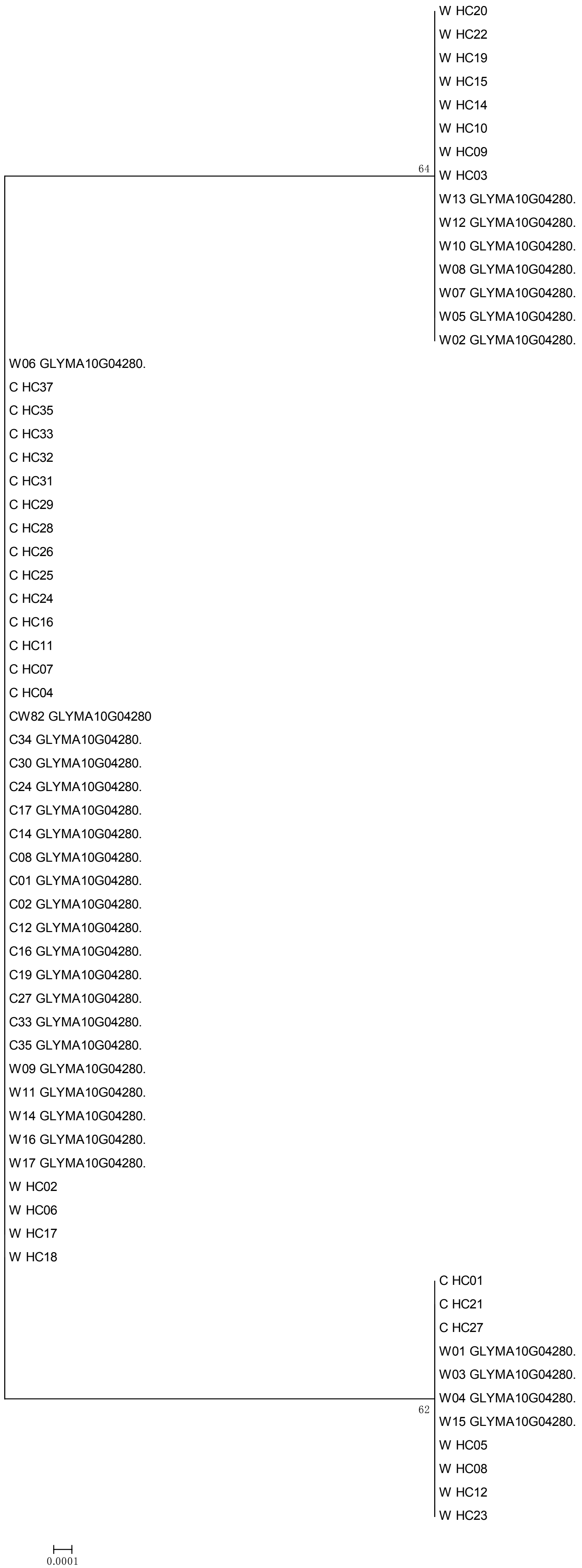
W HC20
W HC22
W HC19
W HC15
W HC14
W HC10
W HC09
64
W HC03
W13 GLYMA10G04280.
W12 GLYMA10G04280.
W10 GLYMA10G04280.
W08 GLYMA10G04280.
W07 GLYMA10G04280.
W05 GLYMA10G04280.
W02 GLYMA10G04280.
W06 GLYMA10G04280.
C HC37
C HC35
C HC33
C HC32
C HC31
C HC29
C HC28
C HC26
C HC25
C HC24
C HC16
C HC11
C HC07
C HC04
CW82 GLYMA10G04280
C34 GLYMA10G04280.
C30 GLYMA10G04280.
C24 GLYMA10G04280.
C17 GLYMA10G04280.
C14 GLYMA10G04280.
C08 GLYMA10G04280.
C01 GLYMA10G04280.
C02 GLYMA10G04280.
C12 GLYMA10G04280.
C16 GLYMA10G04280.
C19 GLYMA10G04280.
C27 GLYMA10G04280.
C33 GLYMA10G04280.
C35 GLYMA10G04280.
W09 GLYMA10G04280.
W11 GLYMA10G04280.
W14 GLYMA10G04280.
W16 GLYMA10G04280.
W17 GLYMA10G04280.
W HC02
W HC06
W HC17
W HC18
C HC01
C HC21
C HC27
W01 GLYMA10G04280.
W03 GLYMA10G04280.
W04 GLYMA10G04280.
62
W15 GLYMA10G04280.
W HC05
W HC08
W HC12
W HC23
0.0001

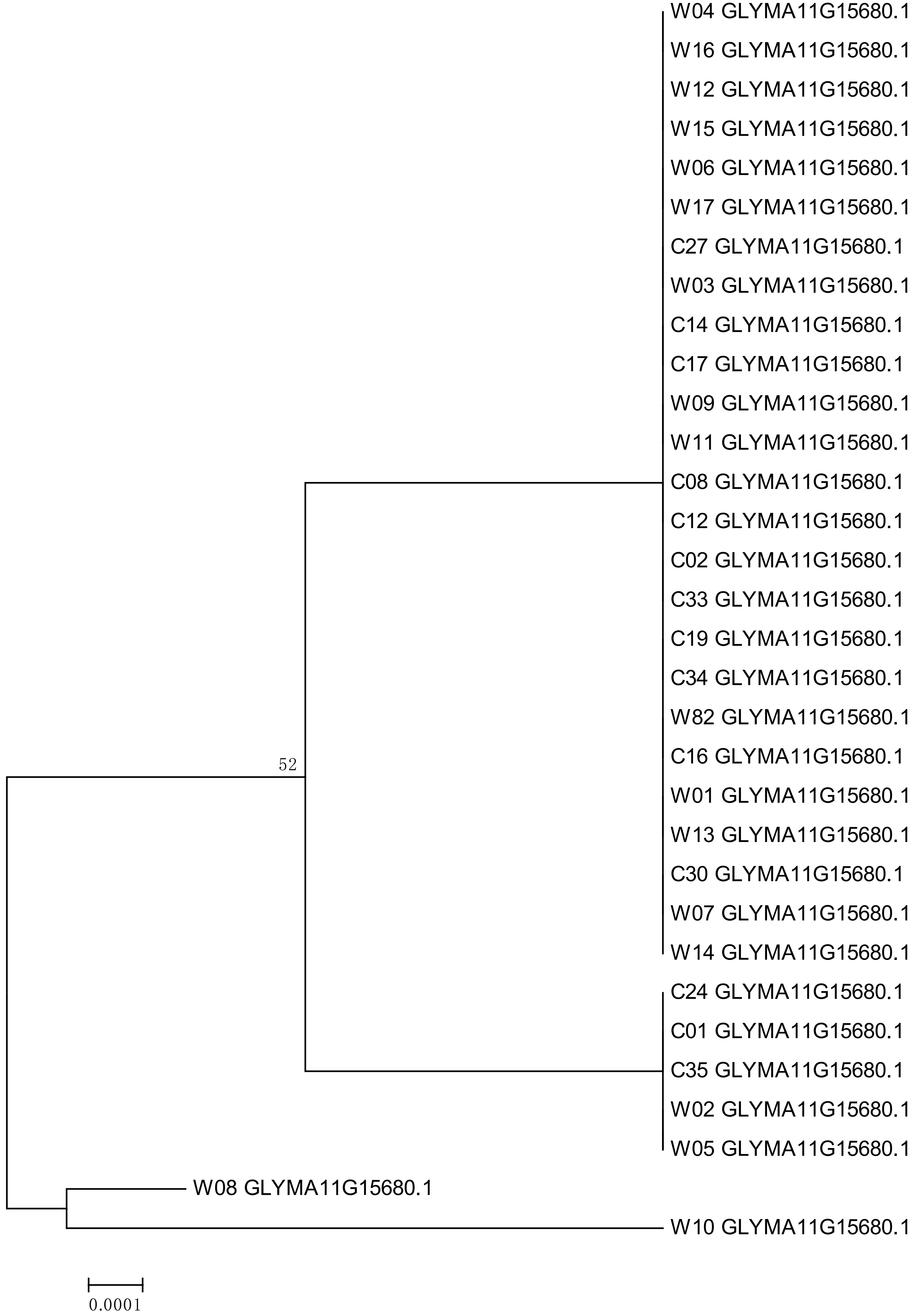
W04 GLYMA11G15680.1
W16 GLYMA11G15680.1
W12 GLYMA11G15680.1
W15 GLYMA11G15680.1
W06 GLYMA11G15680.1
W17 GLYMA11G15680.1
C27 GLYMA11G15680.1
W03 GLYMA11G15680.1
C14 GLYMA11G15680.1
C17 GLYMA11G15680.1
W09 GLYMA11G15680.1
W11 GLYMA11G15680.1
C08 GLYMA11G15680.1
C12 GLYMA11G15680.1
C02 GLYMA11G15680.1
C33 GLYMA11G15680.1
C19 GLYMA11G15680.1
C34 GLYMA11G15680.1
W82 GLYMA11G15680.1
C16 GLYMA11G15680.1
W01 GLYMA11G15680.1
W13 GLYMA11G15680.1
C30 GLYMA11G15680.1
W07 GLYMA11G15680.1
W14 GLYMA11G15680.1
52
C24 GLYMA11G15680.1
C01 GLYMA11G15680.1
C35 GLYMA11G15680.1
W02 GLYMA11G15680.1
W05 GLYMA11G15680.1
W08 GLYMA11G15680.1
W10 GLYMA11G15680.1
0.0001

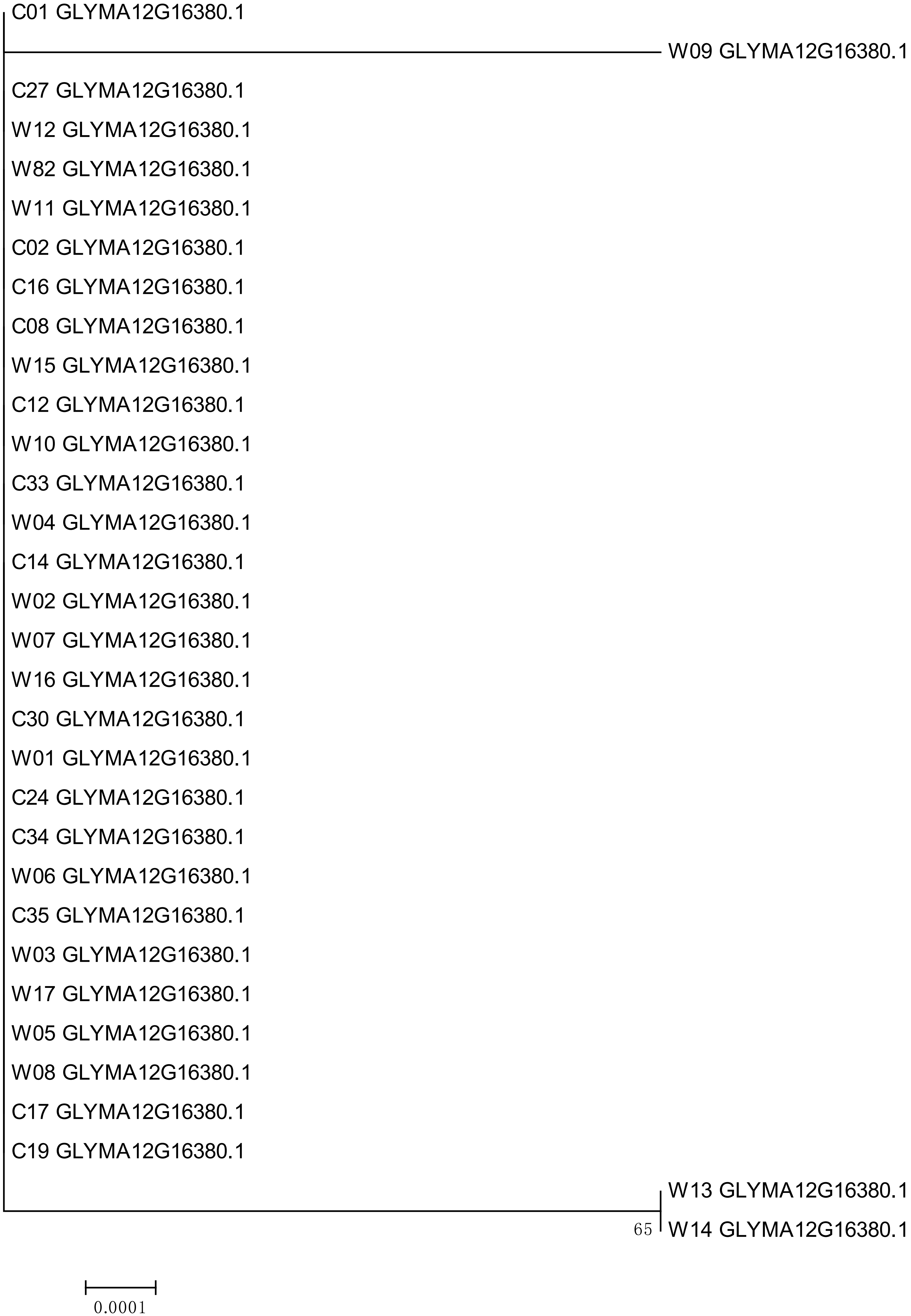
C01 GLYMA12G16380.1
W09 GLYMA12G16380.1
C27 GLYMA12G16380.1
W12 GLYMA12G16380.1
W82 GLYMA12G16380.1
W11 GLYMA12G16380.1
C02 GLYMA12G16380.1
C16 GLYMA12G16380.1
C08 GLYMA12G16380.1
W15 GLYMA12G16380.1
C12 GLYMA12G16380.1
W10 GLYMA12G16380.1
C33 GLYMA12G16380.1
W04 GLYMA12G16380.1
C14 GLYMA12G16380.1
W02 GLYMA12G16380.1
W07 GLYMA12G16380.1
W16 GLYMA12G16380.1
C30 GLYMA12G16380.1
W01 GLYMA12G16380.1
C24 GLYMA12G16380.1
C34 GLYMA12G16380.1
W06 GLYMA12G16380.1
C35 GLYMA12G16380.1
W03 GLYMA12G16380.1
W17 GLYMA12G16380.1
W05 GLYMA12G16380.1
W08 GLYMA12G16380.1
C17 GLYMA12G16380.1
C19 GLYMA12G16380.1
W13 GLYMA12G16380.1
65
W14 GLYMA12G16380.1
0.0001

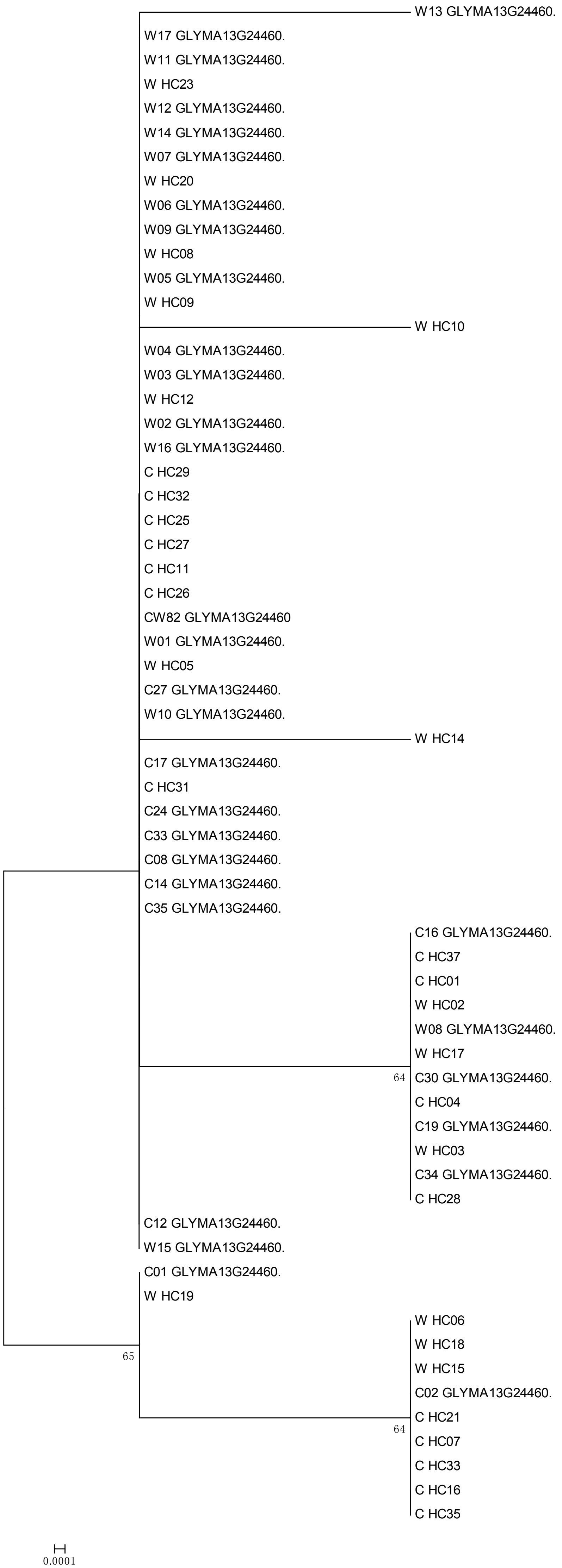

W13 GLYMA13G24460.
W17 GLYMA13G24460.
W11 GLYMA13G24460.
W HC23
W12 GLYMA13G24460.
W14 GLYMA13G24460.
W07 GLYMA13G24460.
W HC20
W06 GLYMA13G24460.
W09 GLYMA13G24460.
W HC08
W05 GLYMA13G24460.
W HC09
W HC10
W04 GLYMA13G24460.
W03 GLYMA13G24460.
W HC12
W02 GLYMA13G24460.
W16 GLYMA13G24460.
C HC29
C HC32
C HC25
C HC27
C HC11
C HC26
CW82 GLYMA13G24460
W01 GLYMA13G24460.
W HC05
C27 GLYMA13G24460.
W10 GLYMA13G24460.
W HC14
C17 GLYMA13G24460.
C HC31
C24 GLYMA13G24460.
C33 GLYMA13G24460.
C08 GLYMA13G24460.
C14 GLYMA13G24460.
C35 GLYMA13G24460.
C16 GLYMA13G24460.
C HC37
C HC01
W HC02
W08 GLYMA13G24460.
W HC17
64
C30 GLYMA13G24460.
C HC04
C19 GLYMA13G24460.
W HC03
C34 GLYMA13G24460.
C HC28
C12 GLYMA13G24460.
W15 GLYMA13G24460.
C01 GLYMA13G24460.
W HC19
65
W HC06
W HC18
W HC15
C02 GLYMA13G24460.
C HC21
64
C HC07
C HC33
C HC16
C HC35
0.0001

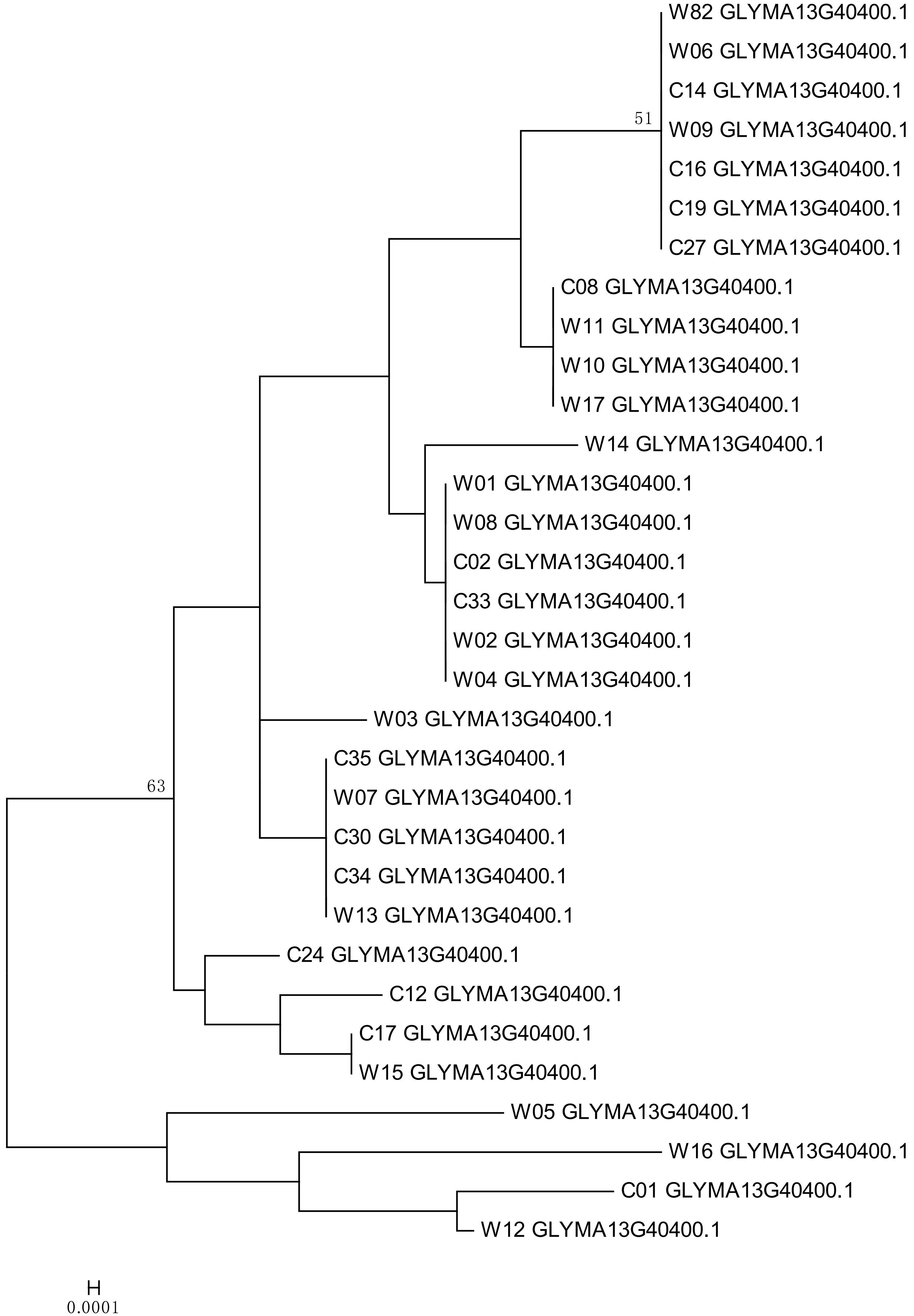

W82 GLYMA13G40400.1
W06 GLYMA13G40400.1
C14 GLYMA13G40400.1
51
W09 GLYMA13G40400.1
C16 GLYMA13G40400.1
C19 GLYMA13G40400.1
C27 GLYMA13G40400.1
C08 GLYMA13G40400.1
W11 GLYMA13G40400.1
W10 GLYMA13G40400.1
W17 GLYMA13G40400.1
W14 GLYMA13G40400.1
W01 GLYMA13G40400.1
W08 GLYMA13G40400.1
C02 GLYMA13G40400.1
C33 GLYMA13G40400.1
W02 GLYMA13G40400.1
W04 GLYMA13G40400.1
W03 GLYMA13G40400.1
C35 GLYMA13G40400.1
63
W07 GLYMA13G40400.1
C30 GLYMA13G40400.1
C34 GLYMA13G40400.1
W13 GLYMA13G40400.1
C24 GLYMA13G40400.1
C12 GLYMA13G40400.1
C17 GLYMA13G40400.1
W15 GLYMA13G40400.1
W05 GLYMA13G40400.1
W16 GLYMA13G40400.1
C01 GLYMA13G40400.1
W12 GLYMA13G40400.1
0.0001

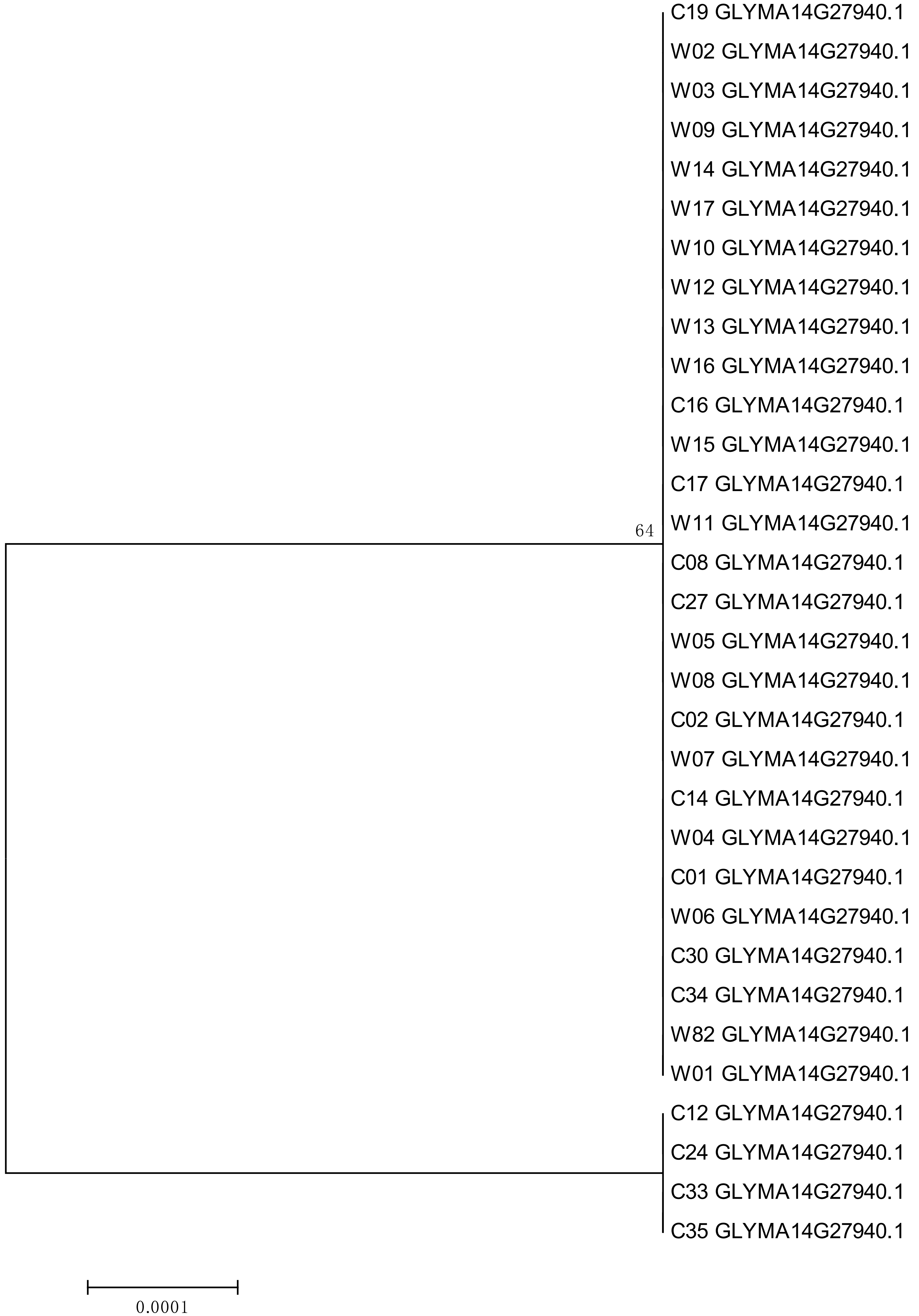

C19 GLYMA14G27940.1
W02 GLYMA14G27940.1
W03 GLYMA14G27940.1
W09 GLYMA14G27940.1
W14 GLYMA14G27940.1
W17 GLYMA14G27940.1
W10 GLYMA14G27940.1
W12 GLYMA14G27940.1
W13 GLYMA14G27940.1
W16 GLYMA14G27940.1
C16 GLYMA14G27940.1
W15 GLYMA14G27940.1
C17 GLYMA14G27940.1
64
W11 GLYMA14G27940.1
C08 GLYMA14G27940.1
C27 GLYMA14G27940.1
W05 GLYMA14G27940.1
W08 GLYMA14G27940.1
C02 GLYMA14G27940.1
W07 GLYMA14G27940.1
C14 GLYMA14G27940.1
W04 GLYMA14G27940.1
C01 GLYMA14G27940.1
W06 GLYMA14G27940.1
C30 GLYMA14G27940.1
C34 GLYMA14G27940.1
W82 GLYMA14G27940.1
W01 GLYMA14G27940.1
C12 GLYMA14G27940.1
C24 GLYMA14G27940.1
C33 GLYMA14G27940.1
C35 GLYMA14G27940.1
0.0001

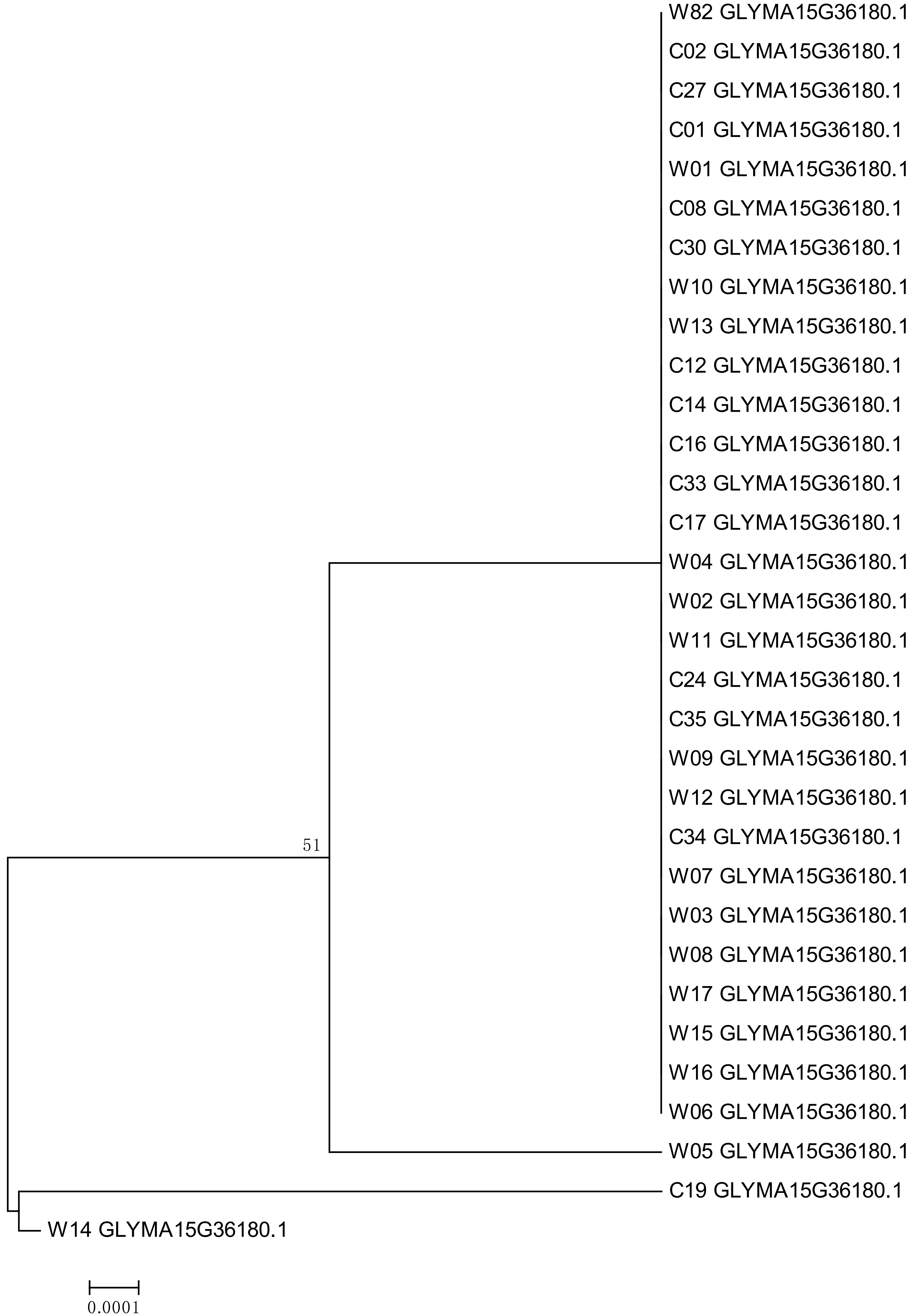
W82 GLYMA15G36180.1
C02 GLYMA15G36180.1
C27 GLYMA15G36180.1
C01 GLYMA15G36180.1
W01 GLYMA15G36180.1
C08 GLYMA15G36180.1
C30 GLYMA15G36180.1
W10 GLYMA15G36180.1
W13 GLYMA15G36180.1
C12 GLYMA15G36180.1
C14 GLYMA15G36180.1
C16 GLYMA15G36180.1
C33 GLYMA15G36180.1
C17 GLYMA15G36180.1
W04 GLYMA15G36180.1
W02 GLYMA15G36180.1
W11 GLYMA15G36180.1
C24 GLYMA15G36180.1
C35 GLYMA15G36180.1
W09 GLYMA15G36180.1
W12 GLYMA15G36180.1
C34 GLYMA15G36180.1
W07 GLYMA15G36180.1
W03 GLYMA15G36180.1
W08 GLYMA15G36180.1
W17 GLYMA15G36180.1
W15 GLYMA15G36180.1
W16 GLYMA15G36180.1
W06 GLYMA15G36180.1
W05 GLYMA15G36180.1
C19 GLYMA15G36180.1
W14 GLYMA15G36180.1
51
0.0001

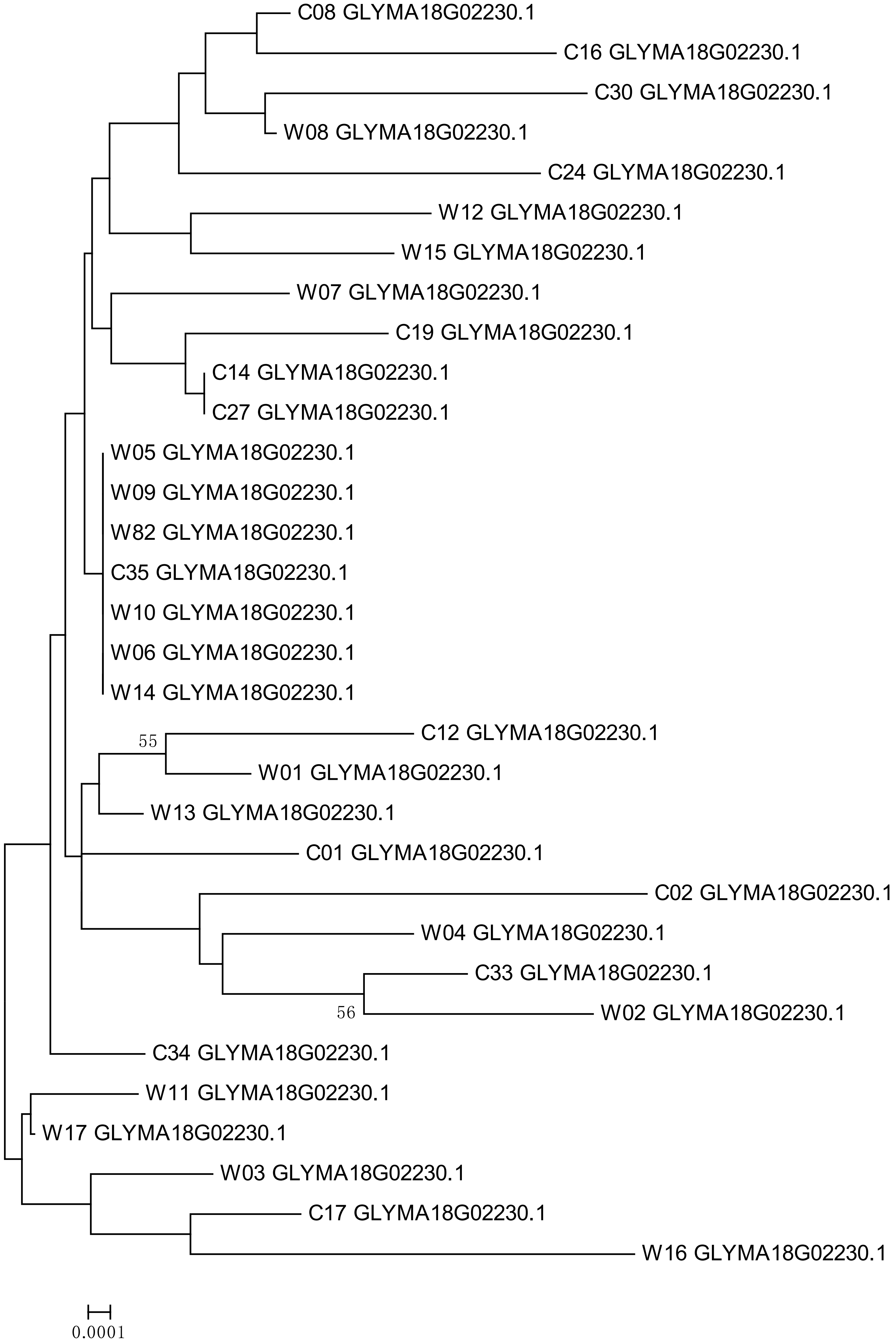
C08 GLYMA18G02230.1
C16 GLYMA18G02230.1
C30 GLYMA18G02230.1
W08 GLYMA18G02230.1
C24 GLYMA18G02230.1
W12 GLYMA18G02230.1
W15 GLYMA18G02230.1
W07 GLYMA18G02230.1
C19 GLYMA18G02230.1
C14 GLYMA18G02230.1
C27 GLYMA18G02230.1
W05 GLYMA18G02230.1
W09 GLYMA18G02230.1
W82 GLYMA18G02230.1
C35 GLYMA18G02230.1
W10 GLYMA18G02230.1
W06 GLYMA18G02230.1
W14 GLYMA18G02230.1
55
C12 GLYMA18G02230.1
W01 GLYMA18G02230.1
W13 GLYMA18G02230.1
C01 GLYMA18G02230.1
C02 GLYMA18G02230.1
W04 GLYMA18G02230.1
C33 GLYMA18G02230.1
56
W02 GLYMA18G02230.1
C34 GLYMA18G02230.1
W11 GLYMA18G02230.1
W17 GLYMA18G02230.1
W03 GLYMA18G02230.1
C17 GLYMA18G02230.1
W16 GLYMA18G02230.1
0.0001

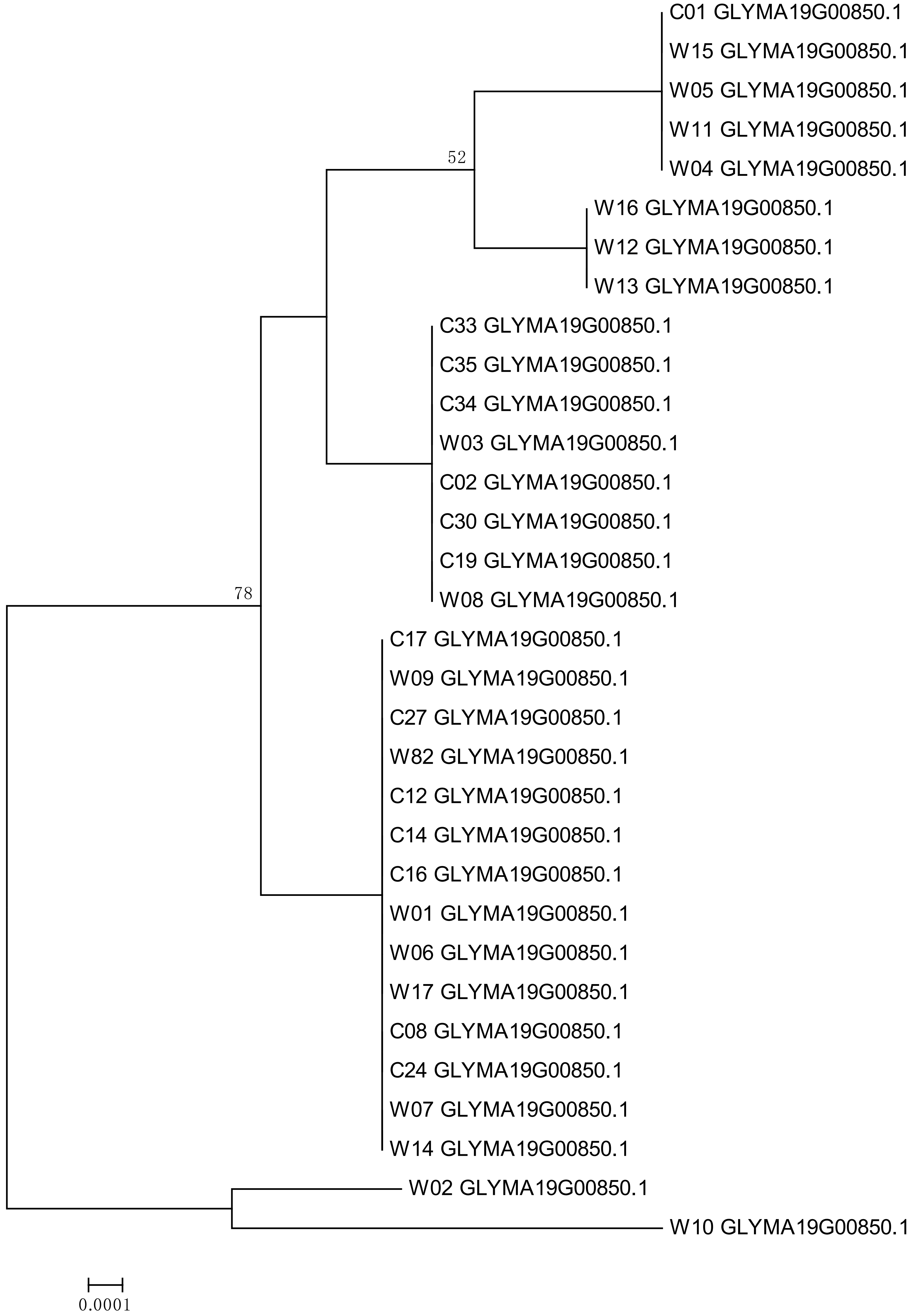
C01 GLYMA19G00850.1
W15 GLYMA19G00850.1
W05 GLYMA19G00850.1
W11 GLYMA19G00850.1
W04 GLYMA19G00850.1
52
W16 GLYMA19G00850.1
W12 GLYMA19G00850.1
W13 GLYMA19G00850.1
C33 GLYMA19G00850.1
C35 GLYMA19G00850.1
C34 GLYMA19G00850.1
W03 GLYMA19G00850.1
C02 GLYMA19G00850.1
C30 GLYMA19G00850.1
C19 GLYMA19G00850.1
W08 GLYMA19G00850.1
78
C17 GLYMA19G00850.1
W09 GLYMA19G00850.1
C27 GLYMA19G00850.1
W82 GLYMA19G00850.1
C12 GLYMA19G00850.1
C14 GLYMA19G00850.1
C16 GLYMA19G00850.1
W01 GLYMA19G00850.1
W06 GLYMA19G00850.1
W17 GLYMA19G00850.1
C08 GLYMA19G00850.1
C24 GLYMA19G00850.1
W07 GLYMA19G00850.1
W14 GLYMA19G00850.1
W02 GLYMA19G00850.1
W10 GLYMA19G00850.1
0.0001
