# Supplementary figures and images for: Diversifying Selection on Flavanone 3-Hydroxylase and Isoflavone Synthase Genes in Cultivated Soybean and Its Wild Progenitors
Source: PLoS One. 2013 Jan 16;8(1):e54154. doi: 10.1371/journal.pone.0054154 (PMC3546919; doi:10.1371/journal.pone.0054154)

**Figure S4 (b)**

**
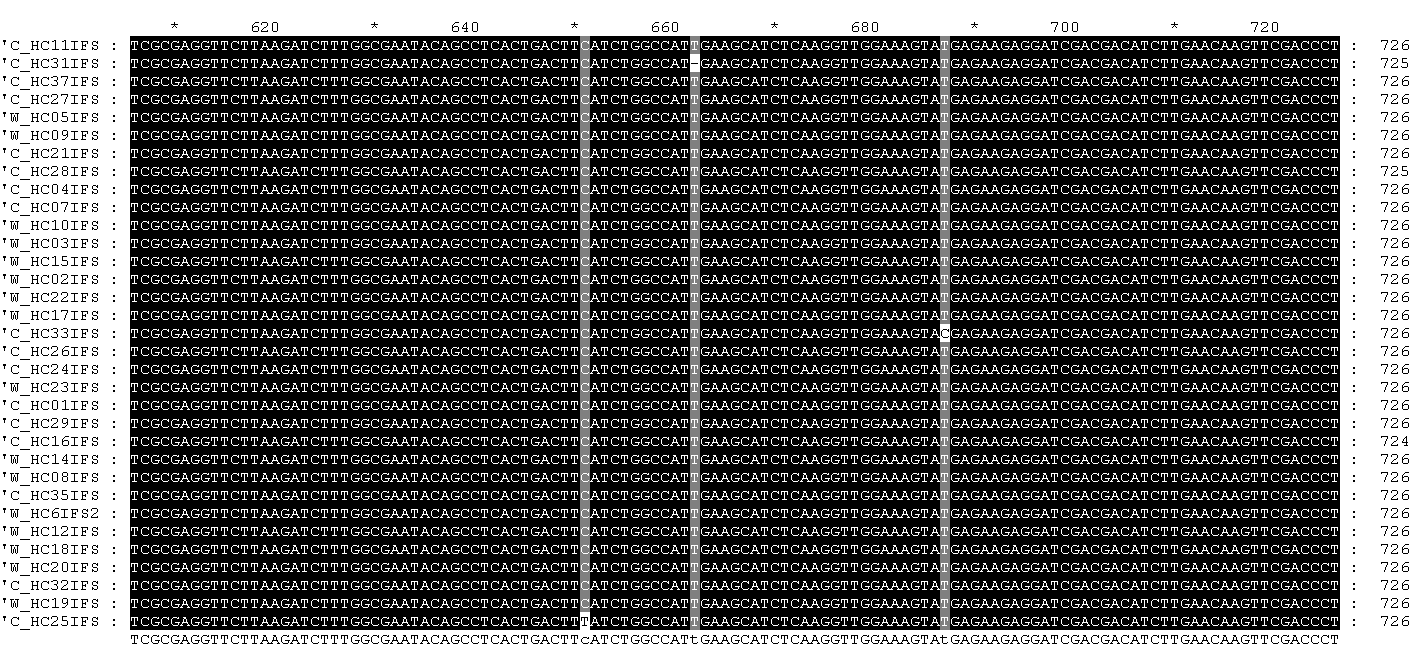
**

**Figure S4 (b)**

**
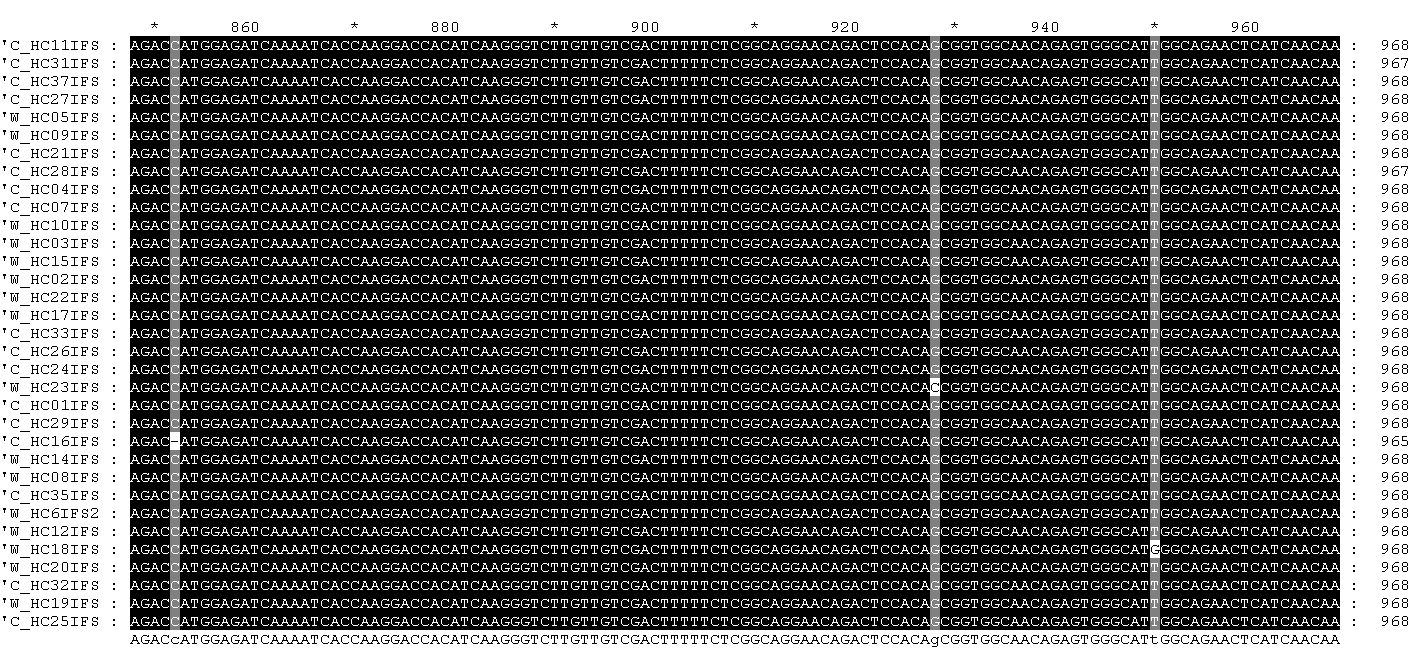
**

Supplement: Figure S4 — (a) Patial (from start codon to 121bp) alignment of IFS1 coding sequence, start codon mutant of accession C_HC35 was shown (red line). (b) Patial aliagnment of IFS2 coding sequence, four indels of 1 or 2bp, two found in accession C_HC16, one in C_HC28 and one in C_HC31, were shown (red arrow). (DOC) [file pone.0054154.s004.doc]
