# Supplementary material for: Diversifying Selection on Flavanone 3-Hydroxylase and Isoflavone Synthase Genes in Cultivated Soybean and Its Wild Progenitors
Source: PLoS One. 2013 Jan 16;8(1):e54154. doi: 10.1371/journal.pone.0054154 (PMC3546919; doi:10.1371/journal.pone.0054154)
Supplement: Table S1 — Thirty-three soybean accessions included in this study. (DOC) [file pone.0054154.s005.doc]

**Table S1.** Thirty-three soybean accessions included in this study

| No. | Accession | Evolution type | Origin ^a^ | Soybean ecological regions in China |
| --- | --- | --- | --- | --- |
| 1 | C_HC01 | *G.max* | Bei’an | I |
| 2 | W_HC02 | *G.soja* | Jixi | I |
| 3 | W_HC03 | *G.soja* | Hailun | I |
| 4 | C_HC04 | *G.max* | Dandong | I |
| 5 | W_HC05 | *G.soja* | Changbai | I |
| 6 | W_HC06 | *G.soja* | Huhehaote | I |
| 7 | C_HC07 | *G.max* | Ji’ning | Ⅱ |
| 8 | W_HC08 | *G.soja* | Huanqu | Ⅱ |
| 9 | W_HC09 | *G.soja* | Pingliang | Ⅱ |
| 10 | W_HC10 | *G.soja* | Huangling | Ⅱ |
| 11 | C_HC11 | *G.max* | Xiantao | Ⅲ |
| 12 | W_HC12 | *G.soja* | Chongyang | Ⅲ |
| 13 | W_HC14 | *G.soja* | Liangping | Ⅲ |
| 14 | W_HC15 | *G.soja* | Xiangyin | Ⅲ |
| 15 | C_HC16 | *G.max* | Zigong | Ⅳ |
| 16 | W_HC17 | *G.soja* | Liancheng | Ⅳ |
| 17 | W_HC18 | *G.soja* | Fengxin | Ⅳ |
| 18 | W_HC19 | *G.soja* | Zixi | Ⅳ |
| 19 | W_HC20 | *G.soja* | Suining | Ⅳ |
| 20 | C_HC21 | *G.max* | Guiyang | Ⅴ |
| 21 | W_HC22 | *G.soja* | Rongjiang | Ⅴ |
| 22 | W_HC23 | *G.soja* | Ninglianglaluo | Ⅴ |
| 23 | C_HC24 | *G.max* | Lufeng | VI |
| 24 | C_HC25 | *G.max* | Qiongshan | VI |
| 25 | C_HC26 | *G.max* | Qionghai | VI |
| 26 | C_HC27 | *G.max* | Beijing | Ⅱ |
| 27 | C_HC28 | *G.max* | Nanjing | Ⅲ |
| 28 | C_HC29 | *G.max* | Nanjing | Ⅲ |
| 29 | C_HC31 | *G.max* | Ji’nan | Ⅱ |
| 30 | C_HC32 | *G.max* | Nanning | VI |
| 31 | C_HC33 | *G.max* | Beijing | Ⅱ |
| 32 | C_HC35 | *G.max* | Huaiyin | Ⅲ |
| 33 | C_HC37 | *G.max* | Nanjing | Ⅲ |

a: The listed are all Chinese cities.
