# Supplementary material for: Diversifying Selection on Flavanone 3-Hydroxylase and Isoflavone Synthase Genes in Cultivated Soybean and Its Wild Progenitors
Source: PLoS One. 2013 Jan 16;8(1):e54154. doi: 10.1371/journal.pone.0054154 (PMC3546919; doi:10.1371/journal.pone.0054154)
Supplement: Table S2 — GenBank accession numbers, functional description and Gene Ontology for reference genes. (DOC) [file pone.0054154.s006.doc]

**Table S2 GenBank accession numbers, functional description and Gene Ontology for reference genes**

| GenBank accession no. | Description | Gene Ontology |
| --- | --- | --- |
| K00821 | lectin (Le1) | sugar binding |
| EU450800 | disease resistance protein Rps1-k-1 | defense response |
| AF083880 | Alternative oxidase precursor (Aox 1) | alternative oxidase activity |
| AF124148 | trehalase 1 GMTRE1 | alpha-trehalase activity |
| U13987 | inducible nitrate reductase 2 (INR2) | nitrate reductase (NADH) activity |
| AF089850 | Urate-degrading peroxidase (PP1) | peroxidase activity |
| E00532 | Heat-shock protein | response to heat |
| M94012 | Maturation-associated protein (MAT9) | response to stress |
| AB004062 | A5A4B3 glycinin | nutrient reservoir activity |
| L10292 | Ascorbate peroxidase | peroxidase activity |
| AB030491 | SG-02 gene for thiamin biosynthetic enzyme | thiamine biosynthetic process |
| M11317 | Low MW heat shock protein | response to stress |
| L20310 | Nodulin (nod-20) | nodulation |
| AF079058 | Alcohol dehydrogenase Adh-1 | alcohol dehydrogenase (NAD) activity |
| D31700 | Cysteine proteinase inhibitor | cysteine-type endopeptidase inhibitor activity |
| D13505 | Early nodulin | nodulation |
| J01298 | actin(SAc1) | ATP binding |
