# Supplementary material for: Diversifying Selection on Flavanone 3-Hydroxylase and Isoflavone Synthase Genes in Cultivated Soybean and Its Wild Progenitors
Source: PLoS One. 2013 Jan 16;8(1):e54154. doi: 10.1371/journal.pone.0054154 (PMC3546919; doi:10.1371/journal.pone.0054154)
Supplement: Table S3 — Genetic differentiation between soybean accessions in our study and the re-sequencing population. (DOC) [file pone.0054154.s007.doc]

**Table S3** Genetic differentiation between soybean accessions in our study and the re-sequencing population.

| Gene | S_nn_ | F_st_ | Chi-square statistic | | |
| --- | --- | --- | --- | --- | --- |
|  |  |  | χ^2^ | df | P-value |
| AF089850 | 0.48 | -0.02 | 1.06 | 2 | 0.59 |
| M94012 | 0.51 | 0.01 | 4.21 | 3 | 0.24 |
| AB004062 | 0.48 | -0.02 | 1.1 | 2 | 0.58 |
| M11317 | 0.53* | 0.08* | 12.03 | 6 | 0.06 |
