# Supplementary material for: Diversifying Selection on Flavanone 3-Hydroxylase and Isoflavone Synthase Genes in Cultivated Soybean and Its Wild Progenitors
Source: PLoS One. 2013 Jan 16;8(1):e54154. doi: 10.1371/journal.pone.0054154 (PMC3546919; doi:10.1371/journal.pone.0054154)
Supplement: Table S4 — Primer information for IFS1 , IFS2 and F3H2 gene and four reference genes. (DOC) [file pone.0054154.s008.doc]

**Table S4** Primer information for *IFS1*, *IFS2* and *F3H2* gene and four reference genes

| Gene | Primers(Upper / Lower) | Annealing temperature (ºC) |
| --- | --- | --- |
| *IFS1* | ATGTGTTTCTGGGGTTATTG / AGTTGTCGTAAGTGAGGCGTC | 60 |
|  | GACGCCTCACTTACGACAACT / AGAAAAAGTCCTACATACCCA | 56 |
|  | TGGGTATGTAGGACTTTTTCT / ATGTAACCTTAATTACTTGAT | 52 |
| *IFS2* | CAGGCAAAGAGAACCAAAACA / TTTACAGTGGTGGCGTTGGGA | 59 |
|  | TCCCAACGCCACCACTGTAAA / AAACGAAGACAAATGGGAGAT | 58 |
| *F3H2* | AGTTCTTTGCTTTGCCACCG / GCTATGGACAAACGGCTATG | 53 |
| M94012 | GCTGCTGGTGGTTACAG/ CGCAAATTTATTCAAAGACAAC | 55 |
| AB004062 | AAGTGGCAAGAACAACAAGATG/ACGGAAAAATTACTGGTAGATGTGTA | 55 |
| AF089850 | ATGTTTGGCAAGTAGGAA/ ACTGCGATGGCAAGACACTA | 55 |
| M11317 | TTTTTGTTTAAGTTACTGTACT/ AGGCTTAACATCAGACTTCTTA | 55 |
