# Supplementary material for: Diversifying Selection on Flavanone 3-Hydroxylase and Isoflavone Synthase Genes in Cultivated Soybean and Its Wild Progenitors
Source: PLoS One. 2013 Jan 16;8(1):e54154. doi: 10.1371/journal.pone.0054154 (PMC3546919; doi:10.1371/journal.pone.0054154)
Supplement: Table S5 — Nucleotide diversity (%) within and between groups at F3H2. (DOC) [file pone.0054154.s009.doc]

***Table S5*** *Nucleotide diversity (%) within and between groups at F3H2*

| Groups | 1 | 2 | 3 | 4 | 5 |
| --- | --- | --- | --- | --- | --- |
| 1 | 0.123 |  |  |  |  |
| 2 | 0.932** | 0.169 |  |  |  |
| 3 | 1.028** | 0.813* | 0.226 |  |  |
| 4 | 1.794** | 1.466* | 1.023* | 0.264 |  |
| 5 | 3.502** | 3.280** | 2.724* | 2.142** | 0.048 |

** and *** indicate a signiﬁcant difference at *p* < 0.01 and *p* < 0.001 between two groups, respectively; the average nucleotide diversity between groups is calculated from all possible pairs between these groups.
